# Effect of ciprofol–etomidate mixtures for deep sedation during gastrointestinal endoscopy: Protocol for a three-arm, double-blind randomized controlled trial

**DOI:** 10.1371/journal.pone.0350274

**Published:** 2026-06-04

**Authors:** Boxuan Xu, Min Niu, Xueling Zhu, Min Cui, Xiaona An, Tianhao Yu, Sihui Tang, Qiuyu Yang, Junjie Wang, Jiguo Si

**Affiliations:** 1 School of Anesthesiology, Shandong Second Medical University, Weifang, Shandong, China; 2 Department of Anesthesiology, Zibo Central Hospital, Zibo, Shandong, China; 3 Department of Anesthesiology, Zibo Maternal and Child Health Hospital, Zibo, Shandong, China; 4 Department of Anesthesiology, Shandong Medical and Pharmaceutical University, Binzhou, Shandong, China; 5 Department of Pain Management, Zibo Central Hospital, Zibo, Shandong, China; Dilla University, ETHIOPIA

## Abstract

**Introduction:**

Deep sedation for gastrointestinal endoscopy can be achieved using either ciprofol or etomidate, both associated with distinct adverse events such as cardiopulmonary depression, myoclonus, and postoperative nausea and vomiting. This study aims to evaluate the safety and efficacy of two ciprofol–etomidate mixtures at varying volume ratios compared with ciprofol alone in patients undergoing gastrointestinal endoscopy.

**Methods:**

This three-arm prospective study will include 135 participants aged 18–65 years who are scheduled for gastrointestinal endoscopy under deep sedation. Patients will be randomly assigned in a 1:1:1 ratio to receive either ciprofol alone or a ciprofol–etomidate mixture at volume-to-volume ratios of 1:1 or 2:1. The primary outcome is the composite incidence of various adverse events, including hypotension, hypoxemia, bradycardia, tachycardia, injection site pain, myoclonus, and nausea and vomiting. The secondary outcomes include the success rate of sedation, induction time, awakening time, recovery time, vital signs, and patient satisfaction. Analyses will be conducted using intention-to-treat and per-protocol approaches.

**Discussion:**

This three-arm randomized controlled trial will determine the potential benefits of combining ciprofol with etomidate for deep sedation during gastrointestinal endoscopy, with a focus on enhanced cardiopulmonary stability, reduced injection site pain, decreased incidence of myoclonus, and lower rates of nausea and vomiting.

**Trial registration:**

This trial has been registered on ChiCTR.gov.cn (ChiCTR2400093109), on Nov 28, 2024.

## Introduction

Gastrointestinal endoscopy, a minimally invasive procedure, is considered the optimal diagnostic and therapeutic approach for gastrointestinal disorders [1,2]. Advancements in painless techniques and their broader applications have significantly increased the number of gastrointestinal endoscopic procedures worldwide over the past decade [3]. Sedation for gastrointestinal endoscopy typically involves a combination of sedatives and analgesics [4,5], and propofol is currently the most widely used sedative due to its rapid onset and offset of action [6]. Despite its well-established benefits, including predictable and stable sedation levels and a favourable safety profile, propofol has evident limitations. These include a narrow therapeutic window [7], dose-dependent cardiopulmonary depression [8], and pain at the injection site [9].

Ciprofol—a novel propofol analogue and 2,6-disubstituted phenol derivative [10]—is 4–5 times more potent than propofol because of its higher affinity for the γ-aminobutyric acid type A receptor, enabling the achievement of comparable sedation levels as propofol at its lower emulsion concentration in the aqueous phase, potentially reducing injection site pain [11]. Notably, several studies have demonstrated that ciprofol induces less injection pain than propofol [5, 12]. In addition, ciprofol exhibits pharmacodynamic and pharmacokinetic properties comparable to those of propofol, including rapid onset of action and recovery [5, 11–13]. However, similar to propofol, ciprofol can cause cardiovascular suppression, leading to significant reductions in blood pressure [5, 14]. Hypotension is a well-recognized factor associated with adverse outcomes, including myocardial injury, renal injury, stroke, and even mortality [15, 16].

Etomidate—a rapid-acting imidazole derivative—offers benefits for general anaesthesia induction, particularly because of its favourable therapeutic index and better haemodynamic and respiratory stability compared with propofol [17]. Etomidate has been identified as a viable sedative agent for procedural sedation in non-operating room environments, demonstrating superior cardiopulmonary stability compared with propofol in these settings [18,19]. However, the primary limitations of etomidate use in such settings include myoclonus, postoperative nausea and vomiting, and injection site pain, rather than adrenal insufficiency concerns [17,20].

A previous study reported better safety and efficacy profiles, with fewer haemodynamic and respiratory complications, of a propofol–etomidate mixture for gastroscopy than propofol alone [21]. Furthermore, a systematic review reported that propofol–etomidate combination reduced myoclonus, intraoperative body movement, and postoperative nausea and vomiting compared with etomidate alone [22]. Considering the complementary effects of propofol and etomidate, using a ciprofol–etomidate mixture for sedation during gastrointestinal endoscopy may mitigate the complications associated with each drug individually, while also lessen injection site pain. We hypothesize that the ciprofol–etomidate mixtures for gastrointestinal endoscopic sedation could offer improved safety, efficacy, and comfort. To the best of our knowledge, no prior studies have investigated the use of ciprofol–etomidate combinations for this purpose. Therefore, this study aims to evaluate the safety and efficacy of two ciprofol–etomidate mixtures, prepared at volume-to-volume ratios of 1:1 and 2:1, for sedation in patients undergoing gastrointestinal endoscopy.

## Methods

### Trial design and study setting

This is a single-center, prospective, randomized, three-arm, double-blind trial that will be conducted at Zibo Central Hospital, a teaching hospital in China. Patients undergoing both upper and lower gastrointestinal endoscopy will be randomly assigned to one of three groups in a 1:1:1 ratio. Sedation will be administered using ciprofol (Group C) or a ciprofol–etomidate mixture at volume-to-volume ratios of 1:1 (Group M1) or 2:1 (Group M2). This trial protocol was prepared in compliance with the Standard Protocol Items: Recommendations for Interventional Trials (SPIRIT) 2013 guidelines [23]. Recruitment was commenced in December 2024 and will conclude in December 2025.

### Participants

Patients scheduled for combined gastroscopy and colonoscopy at Zibo Central Hospital will be screened and recruited for this trial. Eligibility assessments will be conducted at an Anaesthesia Clinic.

The inclusion criteria are: (1) adults aged 18–65 years; (2) scheduled for both diagnostic upper and lower gastrointestinal endoscopy under deep sedation; (3) classified as American Society of Anaesthesiologists (ASA) grade I or II; (4) body mass index (BMI) ≥ 18 and < 30 kg/m^2^; (5) willing to participate in the study and provide written informed consent.

The exclusion criteria are: (1) known allergies to study medications or hypersensitivity to eggs or soy products; (2) uncontrolled or poorly controlled hypertension (systolic blood pressure [SBP] ≥ 180 mmHg and/or diastolic blood pressure ≥ 110 mmHg) or hypotension (SBP < 90 mmHg); (3) obstructive sleep apnea defined by a STOP-BANG score ≥ 3 (Table 1) [24]; (4) severe liver dysfunction (Child-Pugh class B or C), kidney dysfunction (serum creatinine > 2 mg/dL), or heart dysfunction (New York Heart Association classes III and IV); (5) adrenal cortical insufficiency (serum cortisol < 3 mcg/dL); (6) history of epilepsy, neurocognitive disorders, or psychiatric conditions; (7) use of sedatives or hypnotics within the past 3 days; (8) history of general anaesthesia within the past 7 days; (9) pregnancy or breastfeeding; (10) history of alcoholism or drug abuse.

**Table 1:** STOP-BANG screening tool for obstructive sleep apnea.

Areas that indicate OSA	Question
**S = snoring**	**Do you snore loudly (louder than talking or loud enough to be heard through closed doors)?**
**T = tiredness**	**Do you often feel tired, fatigued, or sleepy during daytime?**
**O = observed apnea**	**Has anyone observed you stop breathing during your sleep?**
**P = blood pressure**	**Do you have or are you treated for high blood pressure?**
**B = BMI**	**BMI more than 35 kg/m** ^**2**^ **?**
**A = age**	**Age over 50 years old?**
**N = neck circumference**	**Neck circumference greater than 40 cm?**
**G = gender**	**Are you a man?**

Score 1 point for each positive response

Scoring Interpretation: 0–2 Low Risk; 3–4 Intermediate Risk; 5 or more High Risk.

### Interventions

Gastrointestinal endoscopy will be performed in accordance with the national guidelines of China [25]. In the endoscopy preparation room, a 22-gauge cannula will be inserted into the cephalic vein for intravenous access, followed by the administration of 300–500 mL of lactated Ringer’s solution. After transferring to the endoscopy room, patients will be continuously monitored for non-invasive blood pressure (BP), peripheral oxygen saturation (SpO_2_), respiratory rate (RR), and a 5-lead electrocardiogram (ECG). Heart rate (HR), mean arterial pressure (MAP), SpO_2_, and RR will be measured and recorded at 2-min intervals during the induction period and at 5-min intervals thereafter until patient discharge. Oxygen will be continuously administered through a nasal catheter at a flow rate of 8 L/min until the patient is fully alert. One minute before the sedative administration, 50 mcg of fentanyl will be slowly administered intravenously. Subsequently, sedation will be induced over 30 s using one of the following protocols: 0.16 mL/kg of ciprofol (2.5 mg/mL) for Group C, 0.16 mL/kg of a 1:1 (volume/volume) mixture of ciprofol (1.25 mg/mL) and etomidate (1.0 mg/mL) for Group M1, or 0.16 mL/kg of a 2:1 (volume/volume) mixture of ciprofol (1.67 mg/mL) and etomidate (0.67 mg/mL) for Group M2. A dedicated anesthesia nurse will be responsible for preparing the ciprofol–etomidate mixture. The mixture will be prepared 5 minutes prior to anesthesia induction, placed on a treatment tray at room temperature, and does not require protection from light. It must be used within 30 minutes of preparation; any remaining solution must be discarded in a medical waste bag. The depth of sedation will be assessed every 30 s during induction using the Modified Observer’s Assessment of Alertness/Sedation (MOAA/S) scale (Table 2).

**Table 2:** Modified Observer’s Alertness/Sedation scale (MOAA/S).

Score	Description
5	Responds readily to name spoken in normal tone
4	Lethargic response to name spoken in normal tone
3	Responds only after name is called loudly and/or repeatedly
2	Responds only after mild prodding or shaking
1	Responds only after painful trapezius squeeze
0	No response after painful trapezius squeeze

The gastrointestinal endoscopy procedure will commence once the MOAA/S score is ≤ 1. If the MOAA/S score remains >1, a top-up dose, 1/3 of the initial dosage, will be administered 2 min after administering the initial sedative. Notably, up to two top-up doses will be permitted at 2-min intervals, and if further sedation is required beyond these doses, propofol will be administered as the sole alternative sedative. During the sedation maintenance phase, additional top-up doses (1/3 of the initial dose) may be administered at the discretion of the anaesthesiologist if signs of inadequate sedation are observed.

After the gastrointestinal procedure, patients will be transferred to the recovery room. In the recovery room, a nurse anaesthetist will evaluate the MOAA/S score every minute until the patient is fully alert, defined as achieving a score of MOAA/S=5 in three consecutive assessments. Subsequently, the modified Post Anaesthetic Discharge Scoring System (PADSS; Table 3) will be applied, with a score of ≥9 indicating readiness for discharge [26].

**Table 3:** Modified Post-Anesthetic Discharge Scoring System (PADSS).

Variable	Assessment Interpretation	Score
Vital Signs	BP and HR ± 20% of pre-endoscopy value	2
	BP and HR ± 20%−40% of pre-endoscopy value	1
	BP and HR ± 40% of pre-endoscopy value	0
Ambulation	Steady gait, no dizziness or meets pre-endoscopy level	2
	Requires assistance	1
	Unable to ambulate	0
Nausea and vomiting	No or minimal/treated with p.o. medication	2
	Moderate/treated with parenteral medication	1
	Severe/continues despite treatment	0
Pain	Minimal or no pain (Numerical Analogue Scale = 0–3)	2
	Moderate (Numerical Analogue Scale = 4–6)	1
	Severe (Numerical Analogue Scale = 7–10)	0
Surgical bleeding	None or Minimal (not requiring intervention)	2
	Moderate (1 episode of hematemesis or rectal bleeding)	1
	Severe (≥ 2 episodes of hematemesis or rectal bleeding)	0

Patients’ scoring ≥ 9 for two consecutive measurements are considered fit for discharge home

All sedatives will be administered by a single experienced anaesthetist, and all gastrointestinal procedures will be performed by the same experienced endoscopist. The anaesthetist will be responsible for patient safety throughout the endoscopic procedure and the recovery period. To manage adverse events (AEs), specific interventions will be employed as follows: a MAP decrease of ≥25% from baseline will be treated with 6 mg of ephedrine; an HR of <50 beats per minute will be managed with 0.3 mg of atropine or with additional doses as needed; an HR of ≥120 bpm will be treated with 20 mg of esmolol; hypoxemia (SpO_2_ < 90%) due to airway obstruction will be addressed with a chin lift or jaw thrust manoeuvre, and positive pressure ventilation will be applied in cases of apnea; myoclonus with hypertonia of the neck or trunk, interfering with the procedure, will be managed with propofol as an alternative sedative; and nausea with a score of >5 or instances of vomiting will be treated with 4 mg of ondansetron.

### Outcomes

The primary outcome is the composite incidence of AEs including hypotension, hypertension, bradycardia, tachycardia, hypoxemia, and airway intervention.

Hypotension and hypertension are defined as MAP changes ≥ 25% from baseline. Bradycardia is defined as an HR of < 50 beats per minute or a ≥ 25% decrease from baseline, whereas tachycardia is defined as an HR > 100 beats per minute or a ≥ 25% increase from baseline. Hypoxemia is defined as SpO_2_ < 90%. Airway interventions include chin lifts, jaw-thrust manoeuvres, and positive-pressure ventilation.

Secondary outcomes include: (1) Injection site pain, which will be assessed based on verbal responses and observable behavioural signs such as facial grimacing, arm withdrawal, or tears, using a 4-point scale ranging from 0(no pain) to 3(severe pain) (Table 4) [27]. (2) Myoclonus, which refers to sudden, brief, and involuntary muscle jerks that occur irregularly or rhythmically [28], assessed using a 4-point scale (0, no myoclonus; 1, jerks in one or both hands/feet; 2, jerks in one or both arms/legs; and 3, hypertonia of the neck or trunk) [29]. (3) Postoperative nausea and vomiting. The intensity of nausea will be evaluated in the recovery room using a visual analogue scale (VAS) ranging from 0 (no nausea) to 10 (severe nausea), and the number of vomiting episodes will also be documented. (4) Success rate of sedation, defined as requiring no more than two top-up doses during induction and no alternative sedatives during the procedure. (5) Induction time, measured as the interval from the first administration of the study drug to the point where the MOAA/S score reaches ≤ 1 and endoscopy insertion is successful. (6) Awakening time, defined as the time from the last dose of the study drug to achieving three consecutive MOAA/S scores of 5. (7) Recovery time, measured as the duration from the patient’s transfer to the recovery room to achieving a PADSS score of ≥9. (8) Vital signs, including HR, blood pressure, and SpO_2_, monitored throughout the procedure. (9) Patient satisfaction, assessed using a 5-point Likert scale, where 1 represents very dissatisfied and 5 represents highly satisfied.

**Table 4:** Assessment of pain.

Pain score	Degree of pain	Response
0	None	Negative response to questioning
1	Mild	Pain reported in response to questioning only, without any behavioural signs
2	Moderate	Pain reported in response to questioning and accompanied by a hehavioral sign, or pain reported spontaneously without questioning
3	Severe	Strong vocal response or response accompanied by facial grimacing, arm withdrawal or tears

### Participant timeline

**Fig 1:**
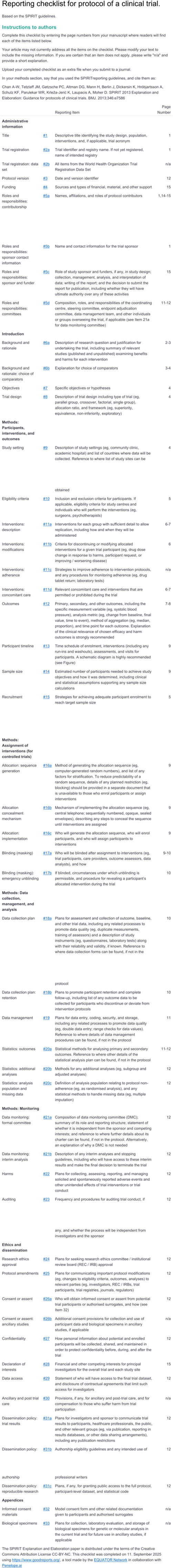
SPIRIT checklist. This completed SPIRIT (Standard Protocol Items: Recommendations for Interventional Trials) checklist indicates the page numbers where each item is addressed in the study protocol.

**Fig 2:**
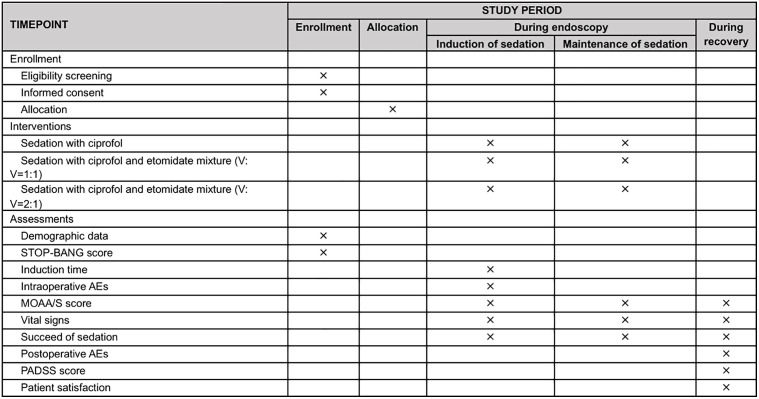
Schedule for enrolment, interventions, and assessments. AEs, adverse events; MOAA/S, modified observer’s assessment of alertness/sedation; PADSS, post anesthetic discharge scoring system; V: V, volume to volume.

**Fig 3:**
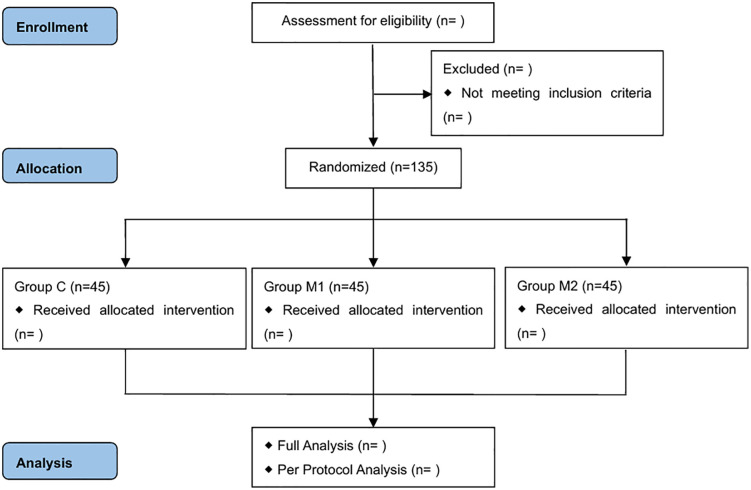
Flowchart of trial design.

The assessments are structured in accordance with the SPIRIT checklist (Fig 1), Fig 2 presents the timeline of participant enrolment, allocation, and interventions. An overview of the study is presented in Fig 3.

### Sample size estimation

A pilot study involving 90 patients revealed composite AEs incidences of 70%, 10%, and 37% in groups C, M1, and M2, respectively (Table 5). Considering a two-sided type I error of 0.05, 80% power, and a 20% dropout rate, the required sample size for this trial was calculated as 138 patients.

**Table 5:** Adverse Events (AEs) in Patients from Groups C, M1, and M2 in the Pilot Study.

	Group C (n = 30)	Group M1(n = 330)	Group M2 (n = 30)
hypotension	17	2	9
hypertension	0	0	0
bradycardia	1	0	0
tachycardia	0	0	0
hypoxemia	2	0	1
airway intervention	1	1	1
Total number of AEs n (%)	21 (70%)	3 (10%)	11 (37%)

### Randomisation and blinding

After providing written informed consent at screening, participants will be randomly assigned to one of the three groups (C, M1, or M2) in a 1:1:1 ratio. An independent biostatistician will generate the allocation sequence utilizing computer-generated random numbers. Allocation details will be concealed in consecutively numbered opaque envelopes. In the examination room, a nurse anaesthetist will open the envelope and prepare one of the following according to the group assignment: ciprofol, a ciprofol–etomidate mixture in a 1:1 volume-to-volume ratio, or a ciprofol–etomidate mixture in a 2:1 volume-to-volume ratio. The appearance of ciprofol and the ciprofol–etomidate mixtures will be indistinguishable. For all groups, the initial dose and top-up dose will be standardised at 0.16 mL/kg and 0.05 mL/kg, respectively. To ensure blinding, the attending anaesthesiologist will not have access to the allocation details. Patients, outcome assessors, and care providers will also remain blinded to group assignments.

A dedicated nurse anaesthetist will be responsible for documenting all safety and efficacy variables, vital signs, and sedative drug administrations from induction to discharge. These data will be recorded in a case report form (CRF), concealing the investigational drug information.

### Data collection

Data collected in this study will be entered into CRFs by researchers who are blinded to the study outcomes and have been trained in data collection and evaluation procedures prior to the research commencement.

Pre-assessment data

1. Demographic details: sex, age, height, weight, BMI.
2. ASA physical status classification.
3. STOP-BANG score.
4. Comorbidities: hypertension, coronary heart disease, diabetes, and respiratory diseases.
5. Electrocardiogram results.
6. Preoperative laboratory tests: blood tests, liver and kidney function tests.

Intraoperative data

1. The baseline vital signs (BP, HR, RR, and SpO_2_) measured after a minimum of 5 min of rest in the endoscopy preparation room.
2. Occurrence of intraoperative AEs, including injection site pain, myoclonus, hypotension, bradycardia, tachycardia, hypoxemia, and positive-pressure ventilation.
3. Vital signs (BP, HR, RR, and SpO_2_) recorded at 2-min intervals during the induction period and 5-min intervals during the maintenance period.
4. Doses of induction and intraoperative top-up study drugs and administration of vasoactive agents.
5. Induction time and awakening time.

Postoperative data

1. Recovery time.
2. Vital signs monitored in the recovery room.
3. Postoperative AEs, including postoperative nausea and vomiting and dizziness.
4. Patient satisfaction, measured using a validated scale.

### Data management

Trial data, including electronic medical records (EMRs), electronic anaesthesia records (EARs), and specially designed electronic CRFs, will be entered directly into a secure database. A password-protected electronic data capture system will be used to ensure participant anonymity. Two months post-trial termination, the data will be uploaded to the ResMAN clinical trial data-sharing platform. Participant files will be retained for 3 years following the completion of the trial.

### Statistical methods

The primary analyses will follow an intention-to-treat approach. Statistical analyses will be conducted using SPSS software (version 25.0; IBM Corporation, Armonk, New York, USA). Qualitative variables will be expressed as totals, percentages, and frequencies and compared using the Pearson Chi-square test or Fisher’s exact test, as appropriate. Quantitative variables will be expressed as the mean ± standard deviation or median (25th and 75th percentiles), depending on the distribution’s normality or non-normality, and analysed using Student’s t-test or the Mann–Whitney U test, as appropriate. Repeated-measures analysis of variance will be employed for vital signs. Statistical significance will be set at p < 0.05. For the primary outcome – the composite incidence of adverse events – a binary logistic regression model will be used to compare the three groups. Odds ratios with 95% confidence intervals will be reported.

### Data monitoring

An independent five-member data monitoring committee (DMC) has been established to oversee this study. The DMC will supervise research implementation and authenticity data. A separate safety-monitoring board will be responsible for monitoring safety outcomes and providing guidance on the management of AEs.

### Ethics and dissemination

The study protocol was approved by the Medical Ethics Committee of Zibo Central Hospital (approval number: 202400194) and registered in the Chinese Clinical Trial Registry (ChiCTR2400093109). Any protocol modifications will be reported to the local institutional review board, trial registry, and the data-monitoring committee. The study will adhere strictly to the principles of the Declaration of Helsinki, and the results will be reported in compliance with the 2010 Consolidated Standards of Reporting Trials (CONSORT) guidelines [30]. The results will be disseminated through peer-reviewed journals and presentations at academic conferences.

### Trial status

The study was conceived and initially designed in 2023, with the current protocol at version 2.0. At the time of manuscript submission, a pilot study has been successfully completed, and we have enrolled 91 participants.

## Discussion

Sedation during gastrointestinal endoscopy is employed to alleviate the patients’ anxiety and discomfort. An ideal sedation protocol minimises the patient’s memory of the procedure while enhancing their cooperation, ultimately improving the quality of the examination and increasing satisfaction for both the patient and physician. Sedation encompasses a spectrum ranging from minimal sedation to general anaesthesia, but the optimal depth and strategy for sedation remain debateable. In China, patients typically prefer deep sedation or general anaesthesia for gastrointestinal endoscopy, commonly achieved through a combination of sedative agents and opioids.

Propofol is currently the most widely used hypnotic agent for sedation and general anaesthesia. Recent studies have demonstrated that ciprofol exhibits similar rapid onset characteristics to propofol but with significantly less injection site pain. However, both propofol and ciprofol are associated with notable cardiopulmonary suppression, which raises concerns, especially for older and frail patients. Hypotension, in particular, is strongly associated with increased morbidity and mortality. A recent perioperative quality initiative consensus statement emphasised that even short periods of systolic blood pressure < 100 mmHg or MAP < 60–70 mmHg can be harmful during non-cardiac surgery [31].

Etomidate, on the other hand, preserves sympathetic tone and myocardial function and causes less apnea than propofol. However, its unfavourable side effects, including a high incidence of myoclonic movements, nausea, and vomiting, limit its use in non-operating room settings. Propofol can mitigate these side effects, including myoclonus and nausea. Several studies have demonstrated the benefits of combining etomidate with propofol for deep sedation during gastrointestinal endoscopy, enhancing patient safety, comfort, and overall satisfaction [21,32,33]. The potential advantages of combining ciprofol with etomidate, such as improved cardiopulmonary stability, reduced injection site pain, decreased myoclonus, and fewer instances of nausea and vomiting, are yet to be fully established. The fundamental challenge in combining these two agents lies in determining whether a mixture can achieve a favourable trade-off between efficacy and toxicity. Therefore, we designed this randomised controlled trial, and to the best of our knowledge, this is the first parallel-design, double-blind trial to evaluate the safety and efficacy of ciprofol–etomidate mixtures for deep sedation during gastrointestinal endoscopy. In this trial, we address this balance through several design features. First, the use of two different volume-to-volume ratios (1:1 and 2:1) allows us to compare not only mixture versus monotherapy but also whether a higher proportion of ciprofol shifts the balance toward better sedation efficacy at the expense of increased hypotension, or vice versa. Second, the composite primary outcome captures a broad spectrum of adverse events—including cardiopulmonary events, myoclonus, and nausea—thereby reflecting overall safety rather than a single toxicity domain. Third, predefined rescue and discontinuation rules ensure that persistent inadequate sedation or intolerable adverse events trigger a switch to propofol, preventing prolonged exposure to an ineffective or unsafe regimen.

### Limitations of the study design

Previous pharmacological research has examined the physical and chemical compatibility of ciprofol–etomidate emulsions, yet the findings remain unpublished, leaving a gap in the evidence base. Additionally, the study’s focus is narrow, as it only investigates the safety and efficacy of 1:1 and 2:1 volume-to-volume ciprofol–etomidate mixtures, leaving out other ratios that might yield different results. Moreover, the study’s population is limited to adults, excluding older and higher-risk endoscopy patients, which restricts the generalizability of the findings to these groups and potentially impacts the applicability of the results in broader clinical contexts.

### Patient and public involvement

No patient or public was involved in the design, conduct, reporting, or dissemination plans of this research.

### Patient consent for publication

Consent obtained directly from patient(s).

## Supporting information

S1 Document Study protocol approved by ethics committee (Chinese version). This document contains the full study protocol reviewed and approved by the ethics committee, written in Chinese.
(DOCX)

S2 Document Study protocol approved by ethics committee (English version). This document contains the full study protocol reviewed and approved by the ethics committee, written in English.
(DOCX)

S3 Document SPIRIT checklist. This completed SPIRIT (Standard Protocol Items: Recommendations for Interventional Trials) checklist indicates the page numbers where each item is addressed in the study protocol.
(DOCX)
