# Supplementary material for: Effect of ciprofol–etomidate mixtures for deep sedation during gastrointestinal endoscopy: Protocol for a three-arm, double-blind randomized controlled trial
Source: PLoS One. 2026 Jun 4;21(6):e0350274. doi: 10.1371/journal.pone.0350274 (PMC13235863; doi:10.1371/journal.pone.0350274)
Supplement: S1 Document — This document contains the full study protocol reviewed and approved by the ethics committee, written in Chinese. (DOCX) [file pone.0350274.s001.docx]

**研究计划书**

**项目名称：环泊酚和依托咪酯混合液用于无痛胃肠镜的效果的研究**

Effect of Ciprofol and Etomidate Mixture for Deep Sedation during Gastrointestinal Endoscopy

**负 责 人：司纪国**

**承担单位：淄博市中心医院**

**电子邮箱：**43254465@qq.com

**研究内容**

一、诚信申明

二、经费来源

三、研究方案

四、研究背景

五、研究假设

六、研究目的

七、研究对象

八、研究设计

九、数据收集

十、研究结果

十一、不良事件

十二、盲法与揭盲

十三、统计学分析

十四、质量控制

十五、可行性分析

十六、经费预算

十七、管理实行

**一、诚信申明**

本课题组郑重声明，本研究所有操作过程均严格按照课题设计方案进行，真实、准确记录试验数据。本课题组所产生的相关研究成果，包括技术标准、专利等相关知识产权归属课题组所有。课题组完全意识到本声明应承担的法律责任。

**二、经费来源**

本项目所需经费由科室自筹。

**三、研究方案**

|  | **研究任务** | **主要目标** |
| --- | --- | --- |
| 2024.10.01-  2024.11.31 | 检索相关研究文献，制定研究计划；医院伦理审查，医学研究登记备案信息系统注册；进行预试验，确定研究方案；制订病例记录与报告表、研究者操作手册。 | **预试验：**纳入30例择期无痛胃肠镜检查的患者，随机分为3组（n=10）：环泊酚组（C组）、环泊酚:依托咪酯（V:V=1:1)组（M1组）、环  泊酚:依托咪酯（V:V=2:1)组（M2组）。记录3组患者围麻醉期不良反应发生率（ 低血压、心动过缓、心动过速、低氧血症、注射部位疼痛、肌阵挛、恶心和呕吐）,通过PASS15.0软件计算样本量。 |
| 2024.12.01-  2025.03.31 | 开展临床试验，完成试验数据采集和录入。 | **临床试验：**根据预试验结果，计算研究所需样本量每组45例。随机纳入135例择期无痛胃肠镜检查的患者，观察并收集指标。 |
| 2025.04.01-  2025.06 .01 | 数据统计分析；撰写课题总结报告。 | **统计分析，撰写论文：**数据整理、统计分析、论文撰写、结题报告等。 |

**四、研究背景**

胃肠道内窥镜检查是一种微创手术，被认为是胃肠道疾病的最佳诊断和治疗方法。在过去的十年里，无痛技术的进步和广泛的应用使得全球胃肠道内窥镜诊疗的数量的大幅增加。丙泊酚由于起效和代谢迅速，是目前无痛胃肠镜中应用最广泛的麻醉药物。然而，丙泊酚也有一些缺点，包括治疗指数小，剂量依赖性心肺抑制、以及注射部位疼痛等。 环泊酚是一种新型的2,6-二取代苯酚衍生物，是丙泊酚的类似物。由于其对γ-氨基丁酸A型受体具有更高的亲和力，其麻醉效能是丙泊酚的4~5倍。这种较高的亲和力允许其在乳化液中以较低的水相的浓度，便可达到与丙泊酚相同的镇静水平，从而减少注射部位的疼痛。一些研究也几经证明，与丙泊酚相比，环泊酚注射疼痛更为轻微。依托咪酯是一种起效、代谢迅速的咪唑衍生物，与丙泊酚相比，它具有良好的治疗指标、血流动力学和呼吸稳定性，对全身麻醉诱导具有潜在的好处。最近有研究将依托咪酯用于手术室外麻醉，认为与丙泊酚相比，依托咪酯有更好的呼吸循环稳定性。然而，在手术室外场景中，限制依托咪酯广泛应用的主要不良反应为肌阵挛、术后恶心和呕吐，以及注射时的疼痛。先前的一项研究表明，在胃镜检查中使用丙泊酚和依托咪酯混合液具有更好的安全性和有效性，与单用丙泊酚相比，血流动力学更平稳，呼吸并发症更少。另外，丙泊酚和依托咪酯的联合使用还可以减少肌阵挛、术中体动反应以及术后恶心呕吐。基于丙泊酚和依托咪酯的互补作用，环泊酚和依托咪酯的混合物用于无痛胃肠镜诊疗麻醉，或许可以减少两种药物单独使用时的相关并发症，并减轻注射疼痛。

**五、研究假设**

我们假设环泊酚和依托咪酯的混合液用于胃肠道内窥镜的镇静可以提供更好的安全性、有效性和患者舒适度。

**六、研究目的**

与单用环泊酚相比，评估两种不同体积比的环泊酚和依托米脂混合液用于无痛胃肠镜检查患者麻醉的安全性和有效性。

**七、研究对象**

研究伦理经淄博市中心医院伦理委员会批准。随机纳入135例拟择期行无痛胃肠镜检查的患者。并依据纳入排除标准完成患者招募。告知患者及家属本临床研究的目的、方法和潜在收益、风险，所有患者或授权家属均签署知情同意书。

⒈ 纳入标准

1. 年龄18~65岁；
2. 择期行无痛胃肠镜检查的患者；
3. ASA分级I-Ⅱ级；
4. 体重指数（BMI）≥18和< 30 kg/m^2^
5. 自愿参加本研究并签署知情同意书。

⒉ 排除标准

(1) 已知对任何一种研究药物过敏，对鸡蛋或豆制品过敏；

(2) 未受控制或控制不良的高血压（收缩压，SBP≥180 mmHg和/或舒张压，DBP≥110 mmHg），或低血压（SBP < 90 mmHg）；

(3) 阻塞性睡眠呼吸暂停（STOP-BANG≥3）；

(4) 严重的肝功能障碍（Child-PughB或C级）、肾功能不全（血清肌酐>2 mg/dL）或心脏功能障碍（纽约心脏协会III和IV级）；

(5) 肾上腺皮质功能不全（血清皮质醇< 3 mcg/dL）；

(6) 癫痫患者、神经认知障碍或精神障碍患者；

(7) 在过去3天内服用镇静剂或催眠药；

(8) 在过去7天内有全身麻醉史；

(9) 怀孕或哺乳期；

(10) 有酗酒或吸毒史。

**八、研究设计**

⒈ 样本量估计

C组、M1组和M2组的综合不良反应发生率分别为60%、50%和20%。以双侧α=0.05的显著水平和1-β=0.9的检验效能，考虑20%的失访率，使用PASS 15.0软件，计算得出该试验总共需要135名患者。

⒉ 随机化

使用随机数字种子采用SPSS软件产生随机数字。

⒊ 试验分组

按照随机数字，按照1:1:1的比例将135例择期行无痛胃肠镜检查的患者随机分为三组：环泊酚组(C组）、环泊酚+依托咪酯体积比1:1混合液组（M1组）、环泊酚+依托咪酯体积比2:1混合液组（M2组）

⒋ 盲法

本试验对参与研究麻醉医师、患者、外科医生、病房医生/护士、以及负责术后资料收集/疗效评价/数据分析的研究者设盲。

⒌ 麻醉干预

⑴麻醉方案

所有接受无痛胃肠镜检查的患者，术前禁食8h，禁水2h。在准备间，均予患者右手背部置入22号留置针，输入300-500 mL乳酸林格氏溶液。进入内镜检查室后，将监测无创血压（BP）、外周血氧饱和度（SpO_2_）、5导联心电图（ECG）和呼吸频率（RR）。

麻醉诱导：在诱导期间，每隔2分钟测量并记录心率（HR）、平均动脉压（MAP）、SpO_2_和RR，然后每隔5分钟记录一次，直到离院。通过鼻导管以8 L/min的流量持续氧气，直到患者完全清醒。在使用镇静剂前1分钟，将缓慢静脉注射50ug芬太尼。随后，C组给予0.16 ml/kg环泊酚（0.4 mg/kg）、M1组0.16ml/kg混合液（环泊酚0.2 mg/kg和依托咪酯0.16mg/kg）、M2组0.16ml/kg混合液（环泊酚0.27 mg/kg和依托咪酯0.11 mg/kg），三组给药时间均超过30 s。在诱导期间，每30秒使用改良观察者的警觉性/镇静评估表（MOAA/S）进行评估。当MOAA/S评分达到≤1时，将开始进行胃肠道内镜检查。如果给药2分钟后MOAA/S评分仍＞1，则追加初始剂量的1/3。每次追加需间隔2 min，最大允许追加次数为2 次。如果需要额外的追加药物，则改行丙泊酚麻醉。

麻醉维持：在麻醉维持阶段，如果出现镇静不足的迹象，麻醉医师将根据经验自行决定每次补充1/3的初始剂量。当胃肠道内镜检查完成，患者将被转运至麻醉恢复室（PACU）进行苏醒。恢复室的麻醉护士将每分钟进行MOAA/S评分，直到患者完全清醒即连续3次MOAA/S=5。随后，将使用麻醉后出院评分系统（PADSS）评分，若PADSS≥9分则表示已经具备出院条件。所有麻醉将由同一位经验丰富的麻醉医师管理，所有胃肠镜检查操作由同一位经验丰富的内镜医生实施 。麻醉医师将在整个检查过程中和恢复期间监测病人，保障病人安全。MAP较基线降低25%，将用6mg麻黄碱治疗；HR ＜50次/分，将根据需要使用0.3~0.5 mg阿托品，可重复使用； HR≥120 bpm，将给予20 mg艾司洛尔；当发生低氧血症（SpO2 < 90%），将采用提下颌手法纠正气道阻塞，使用正压通气处理呼吸暂停。当发生肌阵挛伴颈部或躯干高肌张力，干扰内镜检查时，丙泊酚将作为替代麻醉药物使用。如果恶心评分大于5或发生呕吐，将给予4mg昂丹司琼治疗以缓解症状。

**九、数据收集**

⒈ 检查前数据

1. 基本信息：性别、年龄、身高、体重、ASA分级、术前诊断;
2. 基础生命体征；
3. 既往史：高血压、冠心病、糖尿病等。

⒉ 检查中数据

1. 生命体征：诱导期每2分钟记录生命体征（MAP、HR、SpO_2、_RR）；维持期每5分钟记录生命体征，直至检查结束。
2. 诱导成功时间；
3. 肌颤评分；
4. 是否呼吸道干预；
5. 血管活性药物用量（阿托品、艾司洛尔、麻黄碱等）；
6. 麻醉时间与检查操作时间；
7. 检擦所需镇静药物总量、输入液体总量；

⒊ 术后数据

1. 进入恢复室的时间、每五分钟记录患者生命体征，直至患者离院，
2. 清醒时间；
3. 恢复时间；
4. 有无注射痛；有无恶心、呕吐、头晕；
5. 离院时间。

**十、研究结果**

⒈ 首要结果

各种不良事件（AEs）的综合发生率，包括低血压、高血压、心动过缓、心动过速、低氧血症、气道干预、注射部位疼痛、肌阵挛和术后恶心和呕吐。

⒉ 次要结果

1. 镇静成功率，定义为诱导期间不超过2次补充剂量，整个过程中不需要替代镇静药物；
2. 诱导时间，定义为从第一次给药到MOAA/S≤1和成功插入内镜的时间
3. 清醒时间，定义为从最后一次给药到连续3次MOAA/S评分5的时间；
4. 恢复时间，定义为从运输到恢复室到PADSS评分达到≥9的时间；
5. 整个过程中的生命体征；
6. 患者满意度：1=非常不满意，5=非常满意

**十一、对参试者有效性认定的定义**

⒈ 受试者有权在临床试验的任何阶段随时退出试验。研究者也有义务采取必要措施，包括主动做出让受试者退出临床试验的决定，以保障受试者安全和权益。

⒉ 本研究在以下情况下，研究者应主动考虑让受试者退出临床试验：

⑴ 受试者出现病情加重，继续参与研究将不利于受试者治疗。

⑵ 受试者依从性差，在接受访视、研究干预等方面不能依从临床试验方案执行。

⑶ 出现研究干预相关严重不良反应或不良事件。

⑷ 可能增加受试者风险或损害研究结果可靠性的其他情况。

⒊ 受试者主动退出临床试验

⑴ 受试者不应因此受到任何歧视或报复，其他医疗待遇与权益也不应受到任何影响。

⑵ 受试者退出试验时，应该被告知如何以及从何处获得其他可能的治疗。

⑶ 研究者应尽可能了解受试者主动退出临床试验的原因，并将相关信息记录到原始文件中。

⑷ 受试者退出后的随访应根据方案的要求进行。如果在方案中未明确描述随访要求，可以通过研究团队讨论决定。

⒋ 研究者应该将自己的联系方式主动告知受试者，并主动获取受试者的最新联系方式以确保受试者的按时随访。

⒌ 如果受试者因过敏、不良反应、治疗无效而退出，研究者应根据受试者实际情况积极采取相应的治疗措施。

⒍ 如果受试者因为任何不良事件而退出试验，研究者应该根据方案随访或直到不良事件解决，并将随访的信息记录在原始文件中存档。

⒎ 受试者退出临床试验的相关信息应记录在原始文件中，并定期（比如在年度报告中）提交伦理委员会。

⒏ 研究者在获知或决定受试者退出临床试验后，应完成所有能够完成的评价项目和数据采集。

⒐ 受试者退出试验不意味着已获得的受试者试验数据退出临床试验。截止到受试者退出的时点，已经获得的试验数据应该保留作为试验数据库的一部分提交，不应忽略或者删除。

**十二、不良事件、不良反应的定义及鉴定方法和管理制度**

⒈ 研究者应当以主动的方式，比如与受试者充分交流、主动询问、详尽的体格检查、审核试验室检查数据，充分收集受试者的安全信息，及时准确的判断研究过程中出现的不良事件。

⒉ 不良事件（adverse events，AEs）指患者或临床研究受试者在接受研究干预时发生的不必一定与该治疗干预有因果联系的任何不利的医学事件。不良事件因此可以是与研究干预实施时间上相关的任何不利和意想不到的迹象（如：异常的试验室发现）、症状或疾病，无论是否考虑其与研究干预相关。

⒊ 严重不良事件是指在试验干预任何剂量下或在观察期间任何时候出现的以下不良事件，包括：需延长住院时间、伤残、影响工作和生活、危及生命或死亡、导致先天畸形的事件等。

⒋ 确认不良事件后，应首先作出是否为严重不良事件的判断。一般不良事件根据实际情况，给予相应的临床处理，并填写CRF中的不良事件记录表。

⒌ 不良事件可以明确确认为某种药物不良反应的，依据本中心不良反应报告程序上报。

⒍ 严重不良事件的处理

⑴ 如果严重不良事件威胁到受试者或患者的生命时，需第一时间进行充分的救治，保护受试者的安全，缓解对立情绪。

⑵ 严重不良事件报告

① 必须在首次获悉12h内报告研究单位中心负责人和本中心伦理委员会；

② 在24h内或不迟于第二个工作日向组长单位报告；

③ 做好受试者及其家属的沟通善后工作。

⒎ 不良事件的随访

所有不良反应或事件都应当追踪随访，确认其发展结局，相关事宜至得到妥善解决或病情稳定。

**十三、受试者招募**

⒈ 应针对临床试验方案和研究中心自身的特点制定可行的招募策略，比如针对怎样的潜在受试者人群（如门诊患者/住院患者/志愿者），采用怎样的招募方式（如研究者主动介绍、招募广告）等。

⒉ 必要时，受试者的招募可以指定专门的人员负责，以便与相关工作的管理、信息的记录和进度的把握。

⒊ 无论采取何种方式进行受试者的招募，受试者招募的过程应该是非强制性的。

⒋ 如涉及受试者可以免除的费用，招募中应尽量清晰说明，避免使用“治疗免费”、“化验免费”这样笼统的字眼，以免误会。

⒌ 如果采用发布受试者招募信息的方式获得受试者，招募信息相关材料应经过伦理委员会的审核批准。

⒍ 应特别注意避免招募以下情况的受试者：

⑴ 不在本地居住，难于随访的受试者。

⑵ 因为各种原因，如理解力差、精神失常、语言不通等，难于理解和依从方案的受试者。

⑶ 有导致医患纠纷倾向的受试者。

⑷ 正在参与其他试验研究的受试者。

**十四、盲法与揭盲**

对于每一位参与研究的患者，数据采集完成后检查所有数据以确保其质量并输入数据库，数据库将被锁定并且至揭盲。

1. **统计分析**

初步分析将采用意向治疗（ITT）方法进行。统计分析将使用SPSS软件，25.0版（IBM公司，Armonk，纽约，美国）进行。定性变量将以总数、百分比和频率表示，并酌情使用皮尔逊卡方检验或Fisher精确检验进行比较。定量变量将以均数±标准差或中位数（第25和第75百分位数）表示，这取决于分布的正态性或非正态性，并酌情使用*t*检验或Mann-Whitney *U*检验进行分析。对于生命体征，将采用重复测量的方差分析（ANOVA）。*P*＜ 0.05为具有统计学意义。

**十六、质量管理**

⒈ 参与课题的研究者作为试验流程的实施者和研究数据的提供者，有保证流程实施和研究数据质量的义务；

2. 质量计划

在临床试验启动之前，有必要为课题项目的实施设立一个核心的质量管理小组，并指派专门的质量控制人员；制定质量管理策略，明确质量目标，这一质量管理策略应与如下内容相适应：

⑴ 国际通行的伦理学准则；

⑵ 本地相关适用法规和管理规定；

⑶ 所在机构管理规章；

⑷ 科室内部规章制度；

⑸临床试验方案/SOP。

⒋ 研究团队

根据方案内容分配任务，明确每一位参与研究团队成员的具体分工及职责。建立内部培训机制，确保所有参研人员充分具备完成试验实施所需的知识和信息，并归档培训记录。

⒌ 文件管理

文件记录能帮助研究者对临床试验的实施进行有效的管理，是用于评价试验实施及数据质量的重要依据，集中反映了研究团队成员对管理要求的依从性。团队应该指定专门的人员管理临床研究相关文件。应该建立专门的试验文档，并按时更新，直到监察员审核并确定所有必要的文件均在适当的档案文件夹内，试验才能最后结束。

⒍ 质控人员

⑴ 应指定专门的课题项目质量内审员。

⑵ 质控人员的工作职责请参考本文件“分中心研究团队分工以及岗位职责”质量内审员部分。

⒎ 监察/稽查的配合

⑴ 研究团队内部质量管理工作应与项目监察和稽查体系有机结合、有效配合。

⑵ 在得到通知，接受监察/稽查前，质量内审员有必要对本临床试验项目的实施情况全面审核，发现和处理暴露出来的问题。

⑶ 一般情况下，质量内审员应参与监察/稽查的接待。

⑷ 应详细记录监察/稽查人员发现的问题以及提出的建议和意见，并就存在的问题与监察/稽查人员充分沟通。

⑸ 监察/稽查结束后，应组织或督促召开质量改进会议，听取各方面参研人员就试验实施和存在问题的意见，提出可行的质量改进计划。

**十七、资料管理**

⒈ 定义

⑴ 文件：指描述或记录试验方法、实施流程、试验结果、影响因素以及采取的措施等任何形式（包括且不限于书面、电子、磁性和光学）的记录。

⑵ 文件管理：指临床试验实施过程中保证文件体系的有序和完整的全部活动。

⒉ 基本要求

⑴指定专门的人员管理临床研究相关文件，负责文件的分发、传递、接受、整理、存放和归档。

⑵ 文件管理的周期贯穿临床试验始终。

⑶ 只有经过授权的人员才能查阅临床试验文件，不允许任何未经授权的人员查看文件内容。

⑷ 方案或标准操作流程（standard operation procedure，SOP）规定以外的文件复制必须经过研究负责人的许可。‬‬‬‬‬‬‬‬‬‬‬‬‬‬‬‬

⒊ 文件的集中管理

⑴ 完成记录后的表格性文件应及时集中到文件管理人员处。

⑵ 文件管理人员在接受文件时应第一时间检查文件是否正确、完整和规范，如存在问题应及时处理。

⑶ 文件管理人员在确认文件的正确、完整和规范后，应将文件存放在正确的文件夹中，保存在安全的地方。

4. 文件的存放和归档

⑴ 文件或文件夹一般应保存在带锁的柜中。

⑵ 文件的保存应避免高温、高湿、强光、临近水源和化学试剂，并远离虫患鼠患。

⑶ 存放文件的文件夹应有明显和正确的标示，以便与辨认。

⑷ 应制定文件目录以方便文件的检索和查找。

⑸ 研究相关文件保存时间至少应符合方案和科研管理要求，如上述两方均未提出具体年限要求，应至少在研究结题后保存5年以上。
