# Supplementary material for: Effect of ciprofol–etomidate mixtures for deep sedation during gastrointestinal endoscopy: Protocol for a three-arm, double-blind randomized controlled trial
Source: PLoS One. 2026 Jun 4;21(6):e0350274. doi: 10.1371/journal.pone.0350274 (PMC13235863; doi:10.1371/journal.pone.0350274)
Supplement: S2 Document — This document contains the full study protocol reviewed and approved by the ethics committee, written in English. (DOCX) [file pone.0350274.s002.docx]

Research Proposal

**Project Name:** **Effect of ciprofol–etomidate mixtures for deep sedation during gastrointestinal endoscopy**

# Director: Si Jiguo

**Undertaking unit: Zibo Central Hospital E-mail:** [43254465@qq.com](mailto:43254465@qq.com)

# Research Content

1. Declaration of Integrity 2. Source of Funds

3. Research Plan

1. Research Background
2. Research Hypotheses 6. Research Objectives 7. Research Subjects

8. Research Design 9. Data collection

10. Research Results 11. Adverse events

1. Blinding and unblinding
2. Statistical analysis
3. Quality control
4. Feasibility analysis
5. Budget
6. Management implementation

## 1. Declaration of Integrity

This research group solemnly declares that all operations of this research were carried out strictly in accordance with the project design plan, and the test data were recorded truthfully and accurately. All research results produced by our research group, including technical standards, patents and other related intellectual property rights, belong to our research group. The research group is fully aware of the legal liability of this statement.

## 2. Sources of Funding

The funds required for this project will be self-raised by the department.

## 3. Research Plan

|  | **Research Tasks** | **Main Objectives** |
| --- | --- | --- |
| 2024.10.01 | Retrieve relevant | **Pre-trial:** 30 patients scheduled for elective painless gastroscopy and colonoscopy were included and randomly divided into 3 groups (n=10) : ciprofol group (group C), ciprofol: etomidate (V:V=1:1) group (group M1), and ciprofol: etomidate (V:V=2:1) group (M2 group). The incidence of peri-anesthesia adverse reactions (hypotension, bradycardia, tachycardia, hypoxemia, injection site pain, myoclonus, nausea, and vomiting) was recorded in the three groups, and the sample size was calculated using PASS15.0 software. |
| - | research literature and |  |
| 2024.11.31 | formulate research |  |
|  | plans; Hospital ethics |  |
|  | review, medical |  |
|  | research registration |  |
|  | and filing information |  |
|  | system registration; |  |
|  | Conduct pre-trials and |  |
|  | determine research |  |
|  | protocols; Prepare |  |
|  | case records and |  |
|  | report forms, |  |
|  | researcher operation |  |
|  | manuals. |  |
| 2024.12.01 | Conduct clinical trials | **Clinical trial:** Based on the results of the pre-trial, |
| - | and complete data | calculate the required sample size of 45 cases per group |
| 2025.03.31 | collection and entry | for the study. A total of 135 patients undergoing elective |
|  | for the trials. | painless gastroscopy and colonoscopy were randomly |
|  |  | enrolled to observe and collect indicators. |

| 2025.04.01  -  2025.06.01 | Data statistical analysis; Write the project summary report. | **Statistical analysis, paper writing:** Data organization, statistical analysis, paper writing, final report, etc. |
| --- | --- | --- |

1. **Research Background**

Gastrointestinal endoscopy, a minimally invasive procedure, is considered the best diagnostic and therapeutic approach for gastrointestinal disorders. Advances and widespread application of painless technology over the past decade have led to a significant increase in the number of gastrointestinal endoscopy treatments worldwide. Propofol, due to its rapid onset and metabolism, is currently the most widely used anesthetic in painless gastroscopy and colonoscopy. However, propofol also has some drawbacks, including a low therapeutic index, dose-dependent cardiopulmonary suppression, and pain at the injection site. Ciprofol, a novel 2, 6- disubstituted phenol derivative, is an analogue of propofol. Due to its higher affinity for γ -aminobutyric acid type A receptor, its anesthetic efficacy is 4 to 5 times that of propofol. This higher affinity allows it to achieve the same level of sedation as propofol in emulsions at a lower aqueous concentration, thereby reducing pain at the injection site. Some studies have also shown that ciprofol causes less pain when injected compared to propofol. Etomidate, a fast-acting, metabolized imidazole derivative, has good therapeutic indicators, hemodynamic and respiratory stability compared with propofol, and has potential benefits for general anesthesia induction. Recent studies on the use of etomidate for anesthesia outside the operating room suggest that etomidate has better respiratory and circulatory stability compared to propofol. However, the main adverse reactions that limit the wide use of etomidate in the outside of the operating room are myoclonus, postoperative nausea and vomiting, and pain during injection. A previous study has shown that the use of a mixture of propofol and etomidate during gastroscopy has better safety and efficacy, with more stable hemodynamics and fewer respiratory complications compared to propofol alone. In addition, the combination of propofol and etomidate can also reduce myoclonus, intraoperative body movement reactions, and postoperative nausea and

vomiting. Based on the complementary effects of propofol and etomidate, the use of a mixture of ciprofol and etomidate for anesthesia in painless gastroscopy and colonoscopy may reduce the associated complications when the two drugs are used alone and alleviate injection pain.

## Research Hypotheses

We hypothesize that the mixture of ciprofol and etomidate for sedation in gastrointestinal endoscopy can provide better safety, efficacy and patient comfort.

## 6. Study Objectives

To evaluate the safety and efficacy of two different volume ratios of ciprofol and etomidate mixtures for anesthesia in patients undergoing painless gastroscopy and colonoscopy compared with ciprofol alone.

## Subjects of Study

Research ethics approved by the Ethics Committee of Zibo Central Hospital. 135 patients scheduled for elective painless gastroscopy and colonoscopy were randomly enrolled. And patient recruitment was completed in accordance with inclusion and exclusion criteria. Inform patients and their families of the purpose, methods, potential benefits and risks of this clinical study, and have all patients or authorized family members sign the informed consent form.

1. Inclusion Criteria

1. Age: 18 to 65 years;
2. Patients scheduled for painless gastroscopy and colonoscopy;
3. ASA grade I-II;
4. Body mass index (BMI) ≥18 and < 30 kg/m
5. Voluntary participation in this study and signing of informed consent form.

2 Exclusion Criteria

1. Known allergy to any of the investigational drugs, or allergy to eggs or soy products;
2. Uncontrolled or poorly controlled hypertension (systolic blood pressure, SBP

≥ 180 mmHg and/or diastolic blood pressure, DBP ≥ 110 mmHg), or hypotension (SBP < 90 mmHg);

1. Obstructive sleep apnea (STOP-BANG≥3);
2. Severe liver dysfunction (Child-Pugh B or C grade), renal insufficiency (serum creatinine >2 mg/dL), or cardiac dysfunction (New York Heart Association III and IV grades);
3. Adrenal cortical insufficiency (serum cortisol < 3 mcg/dL);
4. Patients with epilepsy, neurocognitive impairment or mental disorders;
5. Taking sedatives or hypnotics in the past 3 days;
6. Has a history of general anesthesia in the past 7 days;
7. Pregnant or breastfeeding;
8. Has a history of alcohol abuse or drug use.

## Research Design

- - 1. Sample size estimation

The combined incidence rates of adverse reactions in group C, group M1 and group M2 were 60%, 50% and 20%, respectively. With a two-sided significance level of α=0.05 and test power of 1-β=0.9, considering a 20% loss to follow-up rate, using PASS 15.0 software, it was calculated that a total of 135 patients were required for the trial.

1. Randomization

Generate random numbers using the random number seed using SPSS software.

1. Grouping of Trials

According to random numbers, 135 patients scheduled for painless gastroscopy and colonoscopy were randomly divided into three groups at a ratio of 1:1:1: ciprofol group (group C), ciprofol + etomidate 1:1 mixture group (Group M1), and ciprofol + etomidate 2:1 mixture group (group M2).

1. Blinding

This trial blinded anesthesiologists, patients, surgeons, ward doctors/nurses, and researchers responsible for postoperative data collection/efficacy evaluation/data analysis.

5. Anesthesia intervention

(1) Anesthesia plan

All patients undergoing painless gastroscopy and colonoscopy should fast for 8 hours and abstain from water for 2 hours before the operation. In the preparation room, a No. 22 indwelling needle was placed on the back of the right hand of each patient, and 300-500 mL of lactate Ringer's solution was administered. After entering the endoscopy room, non-invasive blood pressure (BP), peripheral blood oxygen saturation (SpO2), 5-lead electrocardiogram (ECG), and respiratory rate (RR) will be monitored.

Anesthesia induction: During induction, heart rate (HR), mean arterial pressure (MAP), SpO2, and RR are measured and recorded every 2 minutes, and then every 5 minutes until discharge. Continuous oxygen was administered through a nasal catheter at a flow rate of 8 L/min until the patient was fully conscious. 50 μ g of fentanyl will be slowly injected intravenously one minute before the sedative is administered. Then, Group C was given 0.16ml/kg ciprofol (0.4 mg/kg), group M1 a 0.16ml/kg mixture (ciprofol 0.2 mg/kg and etomidate 0.16mg/kg), and group M2 a 0.16ml/kg mixture (ciprofol 0.27 mg/kg and etomidate 0.11 mg/kg). The dosing time in all three groups exceeded 30 seconds. During induction, the modified observer's alertness/sedation assessment Scale (MOAA/S) was used every 30 seconds. Gastrointestinal endoscopy will be initiated when the MOAA/S score reaches ≤1. If the MOAA/S score is still > 1 2 minutes after administration, add 1/3 of the initial dose. Each additional dose should be spaced 2 minutes apart, and the maximum number of additional doses allowed is 2. Switch to propofol anesthesia if additional medication is required.

Anesthesia maintenance: During the anesthesia maintenance phase, if there are

signs of insufficient sedation, the anesthesiologist will decide, based on experience, to add 1/3 of the initial dose each time. When the gastrointestinal endoscopy is completed, the patient will be transferred to the anesthesia recovery unit (PACU) for awakening. Anesthesia nurses in the recovery room will score MOAA/S every minute until the patient is fully conscious, that is, MOAA/S=5 for three consecutive times. Subsequently, the post-anesthesia Discharge Scoring System (PADSS) will be used

for scoring, and a PADSS score of 9 or above indicates that the patient is ready for discharge. All anesthesia will be managed by the same experienced anesthesiologist, and all gastroscopy and colonoscopy procedures will be performed by the same experienced endoscopist. The anesthesiologist will monitor the patient throughout the examination and during the recovery period to ensure patient safety. A 25% reduction in MAP from baseline will be treated with 6mg of ephedrine; When HR is less than 50 times per minute, 0.3 to 0.5 mg of atropine will be used as needed, which can be reused; For HR≥120 bpm, 20 mg esmolol will be given; When hypoxemia occurs (SpO2 < 90%), mandibular lifting manipulation will be used to correct airway obstruction, and positive pressure ventilation will be used to treat apnea. Propofol will be used as an alternative anesthetic when myoclonus occurs with high muscle tone in the neck or trunk that interferes with endoscopy. If the nausea score is greater than 5 or vomiting occurs, 4mg of ondansetron will be administered to relieve the symptoms. **9. Data collection**

1. Check the pre-data
2. Basic information: Gender, age, height, weight, ASA classification, preoperative diagnosis;
3. Basic vital signs;
4. Past medical history: Hypertension, coronary heart disease, diabetes, etc.
5. Data from the examination
6. Vital signs: Record vital signs (MAP, HR, SpO2 、 RR) every 2 minutes during the induction period; Vital signs are recorded every 5 minutes during the maintenance period until the examination is completed.
7. Induction success time;
8. Muscle tremor score;
9. Whether respiratory intervention;
10. Dosage of vasoactive drugs (atropine, esmolol, ephedrine, etc.);
11. Anesthesia time and examination operation time;
12. The total amount of sedative drugs required for examination and the total amount of input liquid;

3 Postoperative data

1. Time to enter the recovery room, recording vital signs every five minutes until the patient leaves the hospital,
2. Awakening time;
3. Recovery time;
4. Whether there is injection pain; Whether there is nausea, vomiting or dizziness;
5. Time of discharge.

## 10. Research Results

- 1. Primary Results

The combined incidence of various adverse events (AEs), including hypotension, hypertension, bradycardia, tachycardia, hypoxemia, airway intervention, injection site pain, myoclonus, and postoperative nausea and vomiting.

2 Secondary Outcomes

1. Sedation success rate, defined as no more than two supplementary doses during induction and no need for alternative sedative drugs throughout the process;
2. Induction time is defined as the time from the first administration to MOAA/S≤1 and successful endoscopy insertion
3. Awakening time, defined as the time from the last administration to three consecutive MOAA/S scores of 5;
4. Recovery time, defined as the time from transport to the recovery room to a PADSS score of ≥9;
5. Vital signs throughout the process;
6. Patient satisfaction: 1= very dissatisfied, 5= very satisfied

## 11. Definition of the validity of the participants

1. Subjects have the right to withdraw from the clinical trial at any stage of the trial. Researchers are also obligated to take necessary measures, including proactively making decisions to withdraw subjects from clinical trials, in order to ensure the safety and rights of the subjects.

2 The researcher should proactively consider withdrawing a subject from the clinical trial in the following circumstances:

(1) If a subject's condition worsens, continuing to participate in the study will be detrimental to the treatment of the subject.

⑵ Poor compliance of subjects, unable to comply with the clinical trial protocol in terms of visits, research interventions, etc.

⑶ Serious adverse reactions or events related to the study intervention occurred.

⑷ Other circumstances that may increase the risk to the subjects or undermine the reliability of the study results.

1. The subject voluntarily withdraws from the clinical trial

(1) The subject shall not be subject to any discrimination or retaliation as a result, nor shall any other medical benefits or rights be affected.

⑵ When subjects withdraw from the trial, they should be informed of how and where other possible treatments can be obtained.

⑶ Researchers should know as much as possible the reasons for the subjects' voluntary withdrawal from the clinical trial and record the relevant information in the original document.

⑷ Follow-up after the subjects' withdrawal should be conducted as required by the protocol. If the follow-up requirements are not explicitly described in the protocol, they can be decided through discussion by the research team.

1. Researchers should proactively disclose their contact information to the subjects and proactively obtain the subjects' latest contact information to ensure that the subjects are followed up on time.
2. If a subject withdraws due to an allergy, adverse reaction, or ineffective treatment, the researcher should actively take corresponding treatment measures based on the subject's actual situation.

5. If a subject withdraws from the trial due to any adverse event, the investigator should follow up according to the protocol or until the adverse event is resolved, and record the follow-up information in the original file for archiving.

Youdaoplaceholder0 Information regarding the withdrawal of subjects from

clinical trials should be recorded in the original documents and submitted to the ethics committee on a regular basis (for example, in the annual report).

8 After being informed or decided to withdraw a subject from the clinical trial,

the investigator should complete all available evaluation items and data collection.

Youdaoplaceholder0 the withdrawal of a subject from the trial does not imply the withdrawal of the data obtained from the subject from the clinical trial. As of the time of subject withdrawal, the data obtained from the trial should be retained and submitted as part of the trial database and should not be ignored or deleted.

## 12. Definition, identification methods and management systems of adverse events and adverse reactions

1. Researchers should take an active approach, such as communicating fully with

subjects, asking questions proactively, conducting thorough physical examinations, reviewing laboratory test data, collecting safety information of subjects, and making timely and accurate judgments on adverse events that occur during the study process.

2 adverse events (AEs) refer to any adverse medical event that occurs to a patient

or clinical study subject at the time of the study intervention and does not necessarily have a causal connection with the treatment intervention. Adverse events can therefore be any adverse and unexpected sign (e.g., abnormal laboratory findings), symptom, or disease related to the time of implementation of the study intervention, whether or not their association with the study intervention is considered.

3. Serious adverse events refer to the following adverse events that occur at any

dose of the trial intervention or at any time during the observation period, including: events requiring extended hospital stay, disability, events affecting work and life, events endangering life or death, events causing congenital malformations, etc.

4 After confirming an adverse event, a judgment should be made first as to

whether it is a serious adverse event. For general adverse events, clinical treatment should be given based on the actual situation, and the adverse event record form in the CRF should be filled out.

5. Adverse events that can be clearly identified as adverse drug reactions shall be

reported in accordance with the adverse reaction reporting procedures of this center.

5. Handling of serious adverse events

(1) If a serious adverse event threatens the life of the subject or the patient, adequate treatment should be provided immediately to protect the subject's safety and relieve opposition.

⑵ Reporting of serious adverse events

① Must report to the head of the research unit center and the ethics committee of the center within 12 hours of first knowledge;

② Report to the chair unit within 24 hours or no later than the second working

day;

③ Communicate and handle the aftermath with the subjects and their families. Youdaoplaceholder0 follow-up of adverse events

All adverse reactions or events should be followed up to confirm their

development outcome and related matters until they are properly resolved or the condition is stable.

## Recruitment of subjects

- 1. Feasible recruitment strategies should be developed based on the clinical trial protocol and the characteristics of the research center itself, such as for what potential subject populations (such as outpatients/inpatients/volunteers), and what recruitment methods (such as investigator-initiated referrals, recruitment advertisements), etc.

When necessary, the recruitment of subjects can be assigned to specific personnel to be in charge, so as to facilitate the management of related work, the recording of information and the control of progress.

3 The process of recruiting subjects should be non-mandatory, regardless of the

method used.

1. If it involves fees that can be waived by the subjects, the recruitment should be as clear as possible and avoid using general terms such as "free treatment" or "free laboratory tests" to avoid misunderstandings.
2. If the recruitment of subjects is obtained by Posting the recruitment information, the relevant materials of the recruitment information should be reviewed and approved by the ethics committee.

6 Special attention should be paid to avoiding the recruitment of subjects in the following situations:

(1) Subjects who do not reside locally and are difficult to follow up with.

⑵ Subjects who have difficulty understanding and adhering to the protocol for various reasons, such as poor comprehension, mental disorder, language barrier, etc.

⑶ Subjects with a tendency to cause disputes between doctors and patients.

⑷ Subjects who are participating in other trials.

## Blinding and unblinding

For each patient participating in the study, all data will be checked to ensure its quality and entered into the database after data collection is completed, and the database will be locked and unblinded.

## Statistical analysis

Preliminary analysis will be conducted using intention-to-treat (ITT) methods. Statistical analysis will be conducted using SPSS software version 25.0 (IBM, Armonk, New York, USA). Qualitative variables will be expressed as totals, percentages, and frequencies, and comparisons will be made using Pearson chi-square test or Fisher's exact test as appropriate. Quantitative variables will be expressed as mean ± standard deviation or median (25th and 75th percentiles), depending on the normality or non-normality of the distribution, and analyzed using *t-tests* or Mann- Whitney *U* tests as appropriate. Repeated measures analysis of variance (ANOVA) will be used for vital signs. *P* < 0.05 is considered statistically significant.

## Quality management

1. Researchers participating in the project, as implementers of the trial process and providers of research data, have an obligation to ensure the implementation of the process and the quality of research data;
2. Quality plan

Before the clinical trial is initiated, it is necessary to establish a core quality management team for the implementation of the project and assign dedicated quality control personnel. Develop a quality management strategy with clear quality objectives, which should be adapted to the following:

1. Internationally accepted ethical standards;
2. Local applicable regulations and management rules;

⑶ Regulations governing the institution;

⑷ Internal rules and regulations of the department;

⑸ Clinical trial protocol /SOP.

4 Research Team

Assign tasks based on the content of the plan and clarify the specific division of labor and responsibilities of each member of the research team involved. Establish an internal training mechanism to ensure that all participants are fully equipped with the knowledge and information necessary to complete the trial implementation, and file the training records.

5. Document Management

Documentation helps researchers manage the implementation of clinical trials effectively and is an important basis for evaluating trial implementation and data quality, reflecting the compliance of research team members with management requirements. Teams should appoint dedicated personnel to manage clinical research- related documentation. Specialized trial documentation should be established and updated regularly until the monitor reviews and determines that all necessary documentation is in the appropriate archive folder before the trial is finally concluded.

5. Quality control personnel

1. A dedicated internal auditor for project quality should be appointed.
2. The job responsibilities of the quality control personnel should be referred to the "Sub-center Research Team Division and Job Responsibilities" section on quality internal auditors in this document.

Youdaoplaceholder0 coordination of supervision/inspection

(1) The internal quality management work of the research team should be organically integrated and effectively coordinated with the project supervision and audit system.

⑵ Before being notified to accept the supervision/audit, the internal quality auditor must conduct a comprehensive review of the implementation of this clinical

trial project, identify and address the problems that have been exposed.

Under normal circumstances, quality internal auditors should participate in the reception of supervision/inspection.

⑷ The problems identified by the inspectors and the suggestions and opinions

they put forward should be recorded in detail, and full communication should be made with the inspectors regarding the existing problems.

⑸ After the supervision/inspection, a quality improvement meeting should be

organized or urged to listen to the opinions of all participants on the implementation of the test and the existing problems, and to propose a feasible quality improvement plan.

## Data management

- 1. Definitions

(1) Document: Any form (including but not limited to written, electronic, magnetic, and optical) record that describes or records the test method, process of implementation, test results, influencing factors, and measures taken.

⑵ Document management: All activities that ensure the orderliness and integrity of the document system during the implementation of clinical trials.

2 Basic Requirements

(1) Appoint a specific person to manage the clinical research-related documents, responsible for their distribution, transmission, acceptance, organization, storage and filing.

⑵ The cycle of document management runs throughout the clinical trial.

⑶ Only authorized personnel are allowed to access the clinical trial documents.

No unauthorized personnel are permitted to view the contents of the documents.

⑷ Reproduction of documents other than those specified in the protocol or standard operation procedure (SOP) must be permitted by the study principal.

1. Centralized management of documents

(1) Tabular documents that have been recorded should be promptly brought to the document manager.

⑵ Document managers should check the documents as soon as they receive them to ensure they are correct, complete and standardized. Any problems should be dealt with promptly.

⑶ After confirming that the document is correct, complete and proper, the document manager should store the document in the correct folder and keep it in a safe place.

1. Storage and archiving of documents

(1) Files or folders should generally be kept in locked cabinets.

⑵ Documents should be kept away from high temperatures, high humidity, strong light, near water sources and chemical reagents, and away from pests and rats.

⑶ Folders where files are kept should be clearly and correctly labeled for easy identification.

⑷ A directory of files should be established to facilitate the retrieval and search of files.

⑸ The retention period of research-related documents should be at least in line with the requirements of the program and scientific research management. If neither of the above two parties specifies a specific retention period, they should be kept for at least five years after the conclusion of the research.
