# Supplementary material for: Effect of ciprofol–etomidate mixtures for deep sedation during gastrointestinal endoscopy: Protocol for a three-arm, double-blind randomized controlled trial
Source: PLoS One. 2026 Jun 4;21(6):e0350274. doi: 10.1371/journal.pone.0350274 (PMC13235863; doi:10.1371/journal.pone.0350274)
Supplement: Attachment — Submitted filename: Response to Reviewers.docx [file pone.0350274.s005.docx]

Dear Editor,

We would like to thank you and the reviewers for the thorough and constructive feedback on our manuscript entitled " Effect of ciprofol–etomidate mixtures for deep sedation during gastrointestinal endoscopy: Protocol for a three-arm, double-blind randomized controlled trial " (Manuscript ID: PONE-D-25-50283). The comments have been very helpful in improving the clarity and rigor of our study protocol. We have carefully addressed each point raised and have revised the manuscript accordingly. Below is our point-by-point response to the reviewers' comments.

**Response to Reviewer 1**

**Comment 1:** In this study protocol authors hypothesized that Gusu Constraint Standing Training (GCST) optimizes gait outcome in hemiparetic stroke patients by enhancing their postural adaptation criticality and improving gait control through the common core pathways and nodes shared by posture and gait. But they didn't specify the hypothesized effect sizes. It is thus not clear to me how they managed to calculate the desired sample sizes in this study. Will lost to follow-up be taken into consideration? Currently you designed 7 times points and some initially included patients may not come back so many times. Would the recruitment of 60 hemiparetic stroke patients be sufficient to achieve a meaningful discovery?

**Response:** We sincerely thank the reviewer for their time and thoughtful evaluation of our work. After careful review, we suspect there may have been a minor misunderstanding—possibly the comment pertains to another manuscript—as the content regarding GCST and gait outcomes does not align with our current study, which focuses on sedation regimens for gastrointestinal endoscopy. We are encouraged by the positive remarks in the ‘Responses to Questions’ section and have addressed the reviewer’s points to enhance the quality of our manuscript.

**Response to Reviewer 2**

**Comment 1:** The primary endpoint combines events with very different clinical importance (e.g., injection pain, myoclonus, airway maneuvers, hypoxemia, hemodynamic changes). This can make the study results hard to interpret clinically. Please explain why a composite is appropriate and provide a clear rationale for including each component. Consider focusing on more clinically meaningful safety outcomes (e.g., hypoxemia requiring interventions, hypotension requiring vasopressors).

**Response:** We agree with the reviewer's insightful comment. We have gratefully accepted the reviewer's suggestion to refine our primary endpoint by focusing on more clinically meaningful safety outcomes. Accordingly, injection pain, myoclonus, and postoperative nausea and vomiting have now been reclassified as secondary outcomes. This revision enhances the clinical interpretability of our study findings. This revision has been incorporated into the Outcomes section (Page 8–9, Lines 157–182).

**Comment 2:** The sample size calculation is based on a very small pilot (n=10 per group), which is unstable and may overestimate the effect. I suggest including more patients in the pilot observation or citing published studies. Also, please specify how type I error will be controlled across multiple pairwise comparisons.

**Response:** We thank the reviewer for this valuable comment. Given the absence of comparable prior studies, we extended the pilot phase over the past month and increased the sample size per group to 30 (including the initial 10 per group). This expanded pilot provides more robust and convincing data. Based on the updated pilot results and the newly defined primary outcome, we recalculated the required sample size.

In the pilot, the composite incidence of adverse events in Groups C, M1, and M2 was 70%, 10%, and 37%, respectively. Using multiple tests for two proportions (rather than the Chi-square test initially considered), which helps control the Type I error, the maximum required sample size per group was calculated to be 38. Accounting for a 20% dropout rate, the total sample size required is 138 participants—only one more per group than our original estimate. We plan to retain the target sample size of 135. After the last patient completes the trial, we will calculate the actual dropout rate. If the number of participants eligible for final analysis exceeds 114, we will not re-randomize or adjust the grouping. This information has been added to the Sample size estimation section (Page 9, Lines 188–191).

**Comment 3:** Etomidate-associated myoclonus can lead to unblinding, potentially influencing dosing decisions and airway/hemodynamic interventions. The protocol indicates propofol rescue if myoclonus affects the procedure. However, this altered exposure may directly influence outcomes. Thus, blinding may be compromised and rescue-medication rules may bias outcomes.

**Response:** The reviewer raises a valid methodological concern. The use of etomidate alone for anesthesia induction is associated with a high incidence of myoclonus, whereas combining it with propofol can reduce this adverse effect—this was the rationale behind our design of using a ciprofol–etomidate mixture for total intravenous anesthesia. Although our protocol specified that propofol would be administered as rescue if myoclonus occurred, in our pilot study, no case of severe myoclonus interfered with the completion of gastrointestinal endoscopy.

However, should such a situation arise in the main trial, we plan to include only data collected prior to the rescue intervention (e.g., hypotension, hypoxemia, jaw thrust) in the primary analysis. Data obtained after rescue will be excluded from the primary outcome assessment, as we agree with the reviewer that altered drug exposure may directly influence outcomes. Nonetheless, the incidence of rescue therapy will be recorded and analyzed separately in the final manuscript.

**Comment 4:** The intervention is defined by volume ratios (1:1 and 2:1) without sufficient details. Please provide a detailed preparation procedure (who prepares, aseptic technique, mixing method, labeling, time limits, temperature/light conditions, disposal). Also, report the final concentrations/doses delivered per mL.

**Response:** We thank the reviewer for this valuable suggestion. We acknowledge that this aspect was not clearly described in the original manuscript. In response, we have now added a detailed preparation procedure to the Methods section (Page 6, Lines 126–130). The final concentrations and doses delivered per mL was described in Page 8, Lines 123–126.

**Comment 5:** The restrictive population (age 18–65 years, ASA I–II, BMI 18–30) limits generalizability to higher-risk endoscopy patients.

**Response:** Thank you very much for this valuable suggestion. Focusing on higher-risk endoscopy patients is indeed a clinically important issue that warrants further investigation. Our current study focuses on the effects of a ciprofol–etomidate mixture on respiratory and circulatory depression, as well as adverse events such as injection pain and myoclonus, in a general population aged 18–65 years, with ASA physical status I–II and BMI 18–30. In future research, we plan to explore the impact of this drug combination in patients at high risk of respiratory complications or in older populations. We have added a statement to this effect in the Limitations of the study design section (Page 14, Lines 305–308).

We hope that our revisions and responses adequately address the reviewers' concerns. We believe the manuscript has been significantly improved as a result, and we are grateful for the opportunity to revise our work. We look forward to your further consideration.

Thank you for your time and guidance.

Yours sincerely,
Jiguo Si
[sima225@qq.com](mailto:sima225@qq.com)
