# Supplementary material for: Effect of ciprofol–etomidate mixtures for deep sedation during gastrointestinal endoscopy: Protocol for a three-arm, double-blind randomized controlled trial
Source: PLoS One. 2026 Jun 4;21(6):e0350274. doi: 10.1371/journal.pone.0350274 (PMC13235863; doi:10.1371/journal.pone.0350274)
Supplement: Attachment — Submitted filename: Response_to_Reviewers_auresp_2.docx [file pone.0350274.s006.docx]

Dear Editor,

We would like to thank you and the reviewers for the thorough and constructive feedback on our manuscript entitled " Effect of ciprofol–etomidate mixtures for deep sedation during gastrointestinal endoscopy: Protocol for a three-arm, double-blind randomized controlled trial " (Manuscript ID: PONE-D-25-50283). The comments have been very helpful in improving the clarity and rigor of our study protocol. We have carefully addressed each point raised and have revised the manuscript accordingly. Below is our point-by-point response to the reviewer's comments.

**Response to Reviewer 1**

**Comment 1:** A three-arm follow-up study will be conducted with 135 patients. The sample size calculation is based on an initial pilot data set obtained from the similar population. However, it seems you didn’t adjust for loss to follow up.

**Response:** Thank you for your question. Due to the lack of prior data for reference, we first conducted a pilot study with a small sample size (10 patients per group, 30 in total). A previous reviewer expressed concern about the limited sample size. We have taken this feedback seriously and decided to expand the sample size to 30 patients per group (including the initial 10 per group). Finally, the composite incidence of adverse events in Groups C, M1, and M2 was 70%, 10%, and 37%, respectively. Using multiple tests for two proportions (rather than the Chi-square test initially considered), the maximum required sample size per group was calculated to be 38. Accounting for a 20% dropout rate, this yields 46 patients per group—only one more than our original calculation. Since the study is currently ongoing and randomization has already been performed, we determined not to restart the study. Instead, we will continue enrollment and ensure that at least 38 evaluable patients are obtained per group by the end of the study. The final analysis and report will then be conducted based on these complete data.

**Comment 2:** How the ratio of 1:1 and 2:1 is determined in this study?

**Response:** We thank the reviewer for this valuable comment. Both ciprofol and etomidate act on GABA-A receptors but differ in their potency, pharmacokinetic profiles, and side effects (e.g., injection pain, myoclonus, respiratory depression). Ratios of 1:1 and 2:1 ratio were empirically selected for this preliminary study to compare two fixed-volume mixtures. As this is an initial exploratory protocol, we did not aim to determine the optimal ratio. We have now added a statement in the Discussion acknowledging that further dose-finding studies are needed to establish the best ratio.

**Comment 3:** The discussion on safety management is limited. How do you find a trade-off between toxicity and efficacy?

**Response:** We thank the reviewer for this insightful and important comment. We agree that the original manuscript did not sufficiently discuss how we balance efficacy and toxicity in the study design. In response, we have added a new paragraph in the Discussion section (page 14, line 299-300 and line 303-312) to explicitly address this trade-off. In this newly added part, we clarify as follow.

1. Complementary pharmacology: Ciprofol provides deep sedation with low injection pain but causes hypotension, whereas etomidate offers hemodynamic stability but induces myoclonus and nausea. By combining them in two volume‑to‑volume ratios (1:1 and 2:1), we aim to retain the advantages of each while minimizing their respective toxicities.

2. Composite primary outcome: The use of a composite endpoint (including hypotension, hypoxemia, myoclonus, injection pain, nausea/vomiting, etc.) reflects overall safety rather than a single toxicity, allowing a balanced assessment of net clinical benefit.

3. Rescue and discontinuation rules: Predefined criteria for top‑up doses and alternative sedation (propofol) prevent prolonged exposure to an ineffective or unsafe regimen. Explicit protocols for managing hypotension, bradycardia, hypoxemia, myoclonus and nausea ensure patient safety without compromising the evaluation of the study drugs.

**Comment 4:** The main outcome is the AE incidence. However, in your statistical analysis section you didn’t discuss how to model the treatment effects on this clinical outcome. Are you going to use logistic regression and report odds ratio? Or do you need to use some longitudinal data analysis approaches since you have repeated measures? You mentioned repeated measures ANOVA in this section but this approach may not be suitable for AE incidence.

**Response**: We thank the reviewer for this rigorous and valuable comment. We have revised the Statistical analysis section in the manuscript (page 12, line 251-253) as follows: For the primary outcome—the composite incidence of adverse events—a binary logistic regression model will be used to compare the three groups. Odds ratios with 95% confidence intervals will be reported.

In our original Statistical methods section, we stated that repeated‑measures ANOVA would be employed “for vital signs” rather than primary outcome, there appears to be a misunderstanding regarding the intended use of repeated‑measures ANOVA in our manuscript.

**Comment 5:** Page 3, there is a typesetting error at line 58.

**Response:** We thank the reviewer for pointing out the typesetting error. We have now changed British spelling to American English accordingly. We greatly appreciate the reviewer's careful reading and helpful suggestion.

We hope that our revisions and responses adequately address the reviewer’s concerns. We believe the manuscript has been significantly improved as a result, and we are grateful for the opportunity to revise our work. We look forward to your further consideration.

Thank you for your time and guidance.

Yours sincerely,
Jiguo Si
[sima225@qq.com](mailto:sima225@qq.com)
