# Supplementary material for: Effect of ciprofol–etomidate mixtures for deep sedation during gastrointestinal endoscopy: Protocol for a three-arm, double-blind randomized controlled trial
Source: PLoS One. 2026 Jun 4;21(6):e0350274. doi: 10.1371/journal.pone.0350274 (PMC13235863; doi:10.1371/journal.pone.0350274)
Supplement: Peer review history

# Peer review: Decision Letter 0

**DOI:** 10.1371/journal.pone.0350274.r001

**Authors:** Siraj Ahmed Ali

12 Jan 2026

PONE-D-25-50283

Effect of ciprofol–etomidate mixtures for deep sedation during gastrointestinal endoscopy: Protocol for a three-arm, double-blind randomized controlled trial

PLOS One

Dear Dr. Jiguo Si,

Thank you for submitting your manuscript to PLOS ONE. After careful consideration, we feel that it has merit but does not fully meet PLOS ONE’s publication criteria as it currently stands. Therefore, we invite you to submit a revised version of the manuscript that addresses the points raised during the review process.

Please submit your revised manuscript by Feb 26 2026 11:59PM. If you will need more time than this to complete your revisions, please reply to this message or contact the journal office at plosone@plos.org. When you're ready to submit your revision, log on to https://www.editorialmanager.com/pone/ and select the 'Submissions Needing Revision' folder to locate your manuscript file.

Please include the following items when submitting your revised manuscript:

- A letter that responds to each point raised by the academic editor and reviewer(s). You should upload this letter as a separate file labeled 'Response to Reviewers'.
- A marked-up copy of your manuscript that highlights changes made to the original version. You should upload this as a separate file labeled 'Revised Manuscript with Track Changes'.
- An unmarked version of your revised paper without tracked changes. You should upload this as a separate file labeled 'Manuscript'.

If you would like to make changes to your financial disclosure, please include your updated statement in your cover letter. Guidelines for resubmitting your figure files are available below the reviewer comments at the end of this letter.

If applicable, we recommend that you deposit your laboratory protocols in protocols.io to enhance the reproducibility of your results. Protocols.io assigns your protocol its own identifier (DOI) so that it can be cited independently in the future. For instructions see: https://journals.plos.org/plosone/s/submission-guidelines#loc-laboratory-protocols. Additionally, PLOS ONE offers an option for publishing peer-reviewed Lab Protocol articles, which describe protocols hosted on protocols.io. Read more information on sharing protocols at https://plos.org/protocols?utm_medium=editorial-email&utm_source=authorletters&utm_campaign=protocols.

We look forward to receiving your revised manuscript.

Kind regards,

Siraj Ahmed Ali

Academic Editor

PLOS One

Journal Requirements:

When submitting your revision, we need you to address these additional requirements.

1. Please ensure that your manuscript meets PLOS ONE's style requirements, including those for file naming. The PLOS ONE style templates can be found at

https://journals.plos.org/plosone/s/file?id=wjVg/PLOSOne_formatting_sample_main_body.pdf and

https://journals.plos.org/plosone/s/file?id=ba62/PLOSOne_formatting_sample_title_authors_affiliations.pdf

2. When completing the data availability statement of the submission form, you indicated that you will make your data available on acceptance. We strongly recommend all authors decide on a data sharing plan before acceptance, as the process can be lengthy and hold up publication timelines. Please note that, though access restrictions are acceptable now, your entire data will need to be made freely accessible if your manuscript is accepted for publication. This policy applies to all data except where public deposition would breach compliance with the protocol approved by your research ethics board. If you are unable to adhere to our open data policy, please kindly revise your statement to explain your reasoning and we will seek the editor's input on an exemption. Please be assured that, once you have provided your new statement, the assessment of your exemption will not hold up the peer review process.

3. Please include captions for your Supporting Information files at the end of your manuscript, and update any in-text citations to match accordingly. Please see our Supporting Information guidelines for more information: http://journals.plos.org/plosone/s/supporting-information.

4. If the reviewer comments include a recommendation to cite specific previously published works, please review and evaluate these publications to determine whether they are relevant and should be cited. There is no requirement to cite these works unless the editor has indicated otherwise.

[Note: HTML markup is below. Please do not edit.]

Reviewers' comments:

Reviewer's Responses to Questions

**Comments to the Author**

1. Does the manuscript provide a valid rationale for the proposed study, with clearly identified and justified research questions?

The research question outlined is expected to address a valid academic problem or topic and contribute to the base of knowledge in the field.

Reviewer #1: Partly

Reviewer #2: Yes

**********

2. Is the protocol technically sound and planned in a manner that will lead to a meaningful outcome and allow testing the stated hypotheses?

The manuscript should describe the methods in sufficient detail to prevent undisclosed flexibility in the experimental procedure or analysis pipeline, including sufficient outcome-neutral conditions (e.g. necessary controls, absence of floor or ceiling effects) to test the proposed hypotheses and a statistical power analysis where applicable. As there may be aspects of the methodology and analysis which can only be refined once the work is undertaken, authors should outline potential assumptions and explicitly describe what aspects of the proposed analyses, if any, are exploratory.

Reviewer #1: Partly

Reviewer #2: Yes

**********

3. Is the methodology feasible and described in sufficient detail to allow the work to be replicable?

Descriptions of methods and materials in the protocol should be reported in sufficient detail for another researcher to reproduce all experiments and analyses. The protocol should describe the appropriate controls, sample size calculations, and replication needed to ensure that the data are robust and reproducible.

Reviewer #1: Yes

Reviewer #2: Yes

**********

4. Have the authors described where all data underlying the findings will be made available when the study is complete?

The PLOS Data policy requires authors to make all data underlying the findings described in their manuscript fully available without restriction, with rare exception, at the time of publication. The data should be provided as part of the manuscript or its supporting information, or deposited to a public repository. For example, in addition to summary statistics, the data points behind means, medians and variance measures should be available. If there are restrictions on publicly sharing data—e.g. participant privacy or use of data from a third party—those must be specified.

Reviewer #1: Yes

Reviewer #2: Yes

**********

5. Is the manuscript presented in an intelligible fashion and written in standard English?

PLOS ONE does not copyedit accepted manuscripts, so the language in submitted articles must be clear, correct, and unambiguous. Any typographical or grammatical errors should be corrected at revision, so please note any specific errors here.

Reviewer #1: Yes

Reviewer #2: Yes

**********

6. Review Comments to the Author

Please use the space provided to explain your answers to the questions above and, if applicable, provide comments about issues authors must address before this protocol can be accepted for publication. You may also include additional comments for the author, including concerns about research or publication ethics.

You may also provide optional suggestions and comments to authors that they might find helpful in planning their study.

(Please upload your review as an attachment if it exceeds 20,000 characters)

Reviewer #1: In this study protocol authors hypothesized that Gusu Constraint Standing Training (GCST) optimizes gait outcome in hemiparetic stroke patients by enhancing their postural adaptation criticality and improving gait control through the common core pathways and noeds shared by posture and gait. But they didn’t specify the hypothesized effect sizes.

It is thus not clear to me how they managed to calculate the desired sample sizes in this study. Will lost to follow-up be taken into consideration? Currently you designed 7 times points and some initially included patients may not come back so many times. Would the recruitment of 60 hemiparetic stroke patients be sufficient to achieve a meaningful discovery?

Table 1, is there any reason to differentiate the three t0? Since some measurements are not taken in all three hospitals, you may also need to adjust the site in the model.

The statistical analysis is straightforward and I don’t see any severe issues. But because the sample size is relatively low they may not afford conducting too many tests without proper adjustment of type I errors. Since you have multiple time points, you may also consider a more flexible longitudinal model to obtain multi-step analysis results.

Figure resolutions can be improved. Currently it is very hard for me to read the small and blurring fonts.

Reviewer #2: This is a protocol for a single-center, three-arm, double-blind randomized controlled trial comparing ciprofol alone versus ciprofol–etomidate mixtures (1:1 and 2:1) for sedation during gastrointestinal endoscopy. The randomized, blinded, three-arm design and trial registration are strengths. However, several issues currently limit interpretability and internal validity.

1. The primary endpoint combines events with very different clinical importance (e.g., injection pain, myoclonus, airway maneuvers, hypoxemia, hemodynamic changes). This can make the study results hard to interpret clinically. Please explain why a composite is appropriate and provide a clear rationale for including each component. Consider focusing on more clinically meaningful safety outcomes (e.g., hypoxemia requiring interventions, hypotension requiring vasopressors).

2. The sample size calculation is based on a very small pilot (n=10 per group), which is unstable and may overestimate the effect. I suggest including more patients in the pilot observation or citing published studies. Also, please specify how type I error will be controlled across multiple pairwise comparisons.

3. Etomidate-associated myoclonus can lead to unblinding, potentially influencing dosing decisions and airway/hemodynamic interventions. The protocol indicates propofol rescue if myoclonus affects the procedure. However, this altered exposure may directly influence outcomes. Thus, blinding may be compromised and rescue-medication rules may bias outcomes.

4. The intervention is defined by volume ratios (1:1 and 2:1) without sufficient details. Please provide a detailed preparation procedure (who prepares, aseptic technique, mixing method, labeling, time limits, temperature/light conditions, disposal). Also, report the final concentrations/doses delivered per mL.

5. The restrictive population (age 18–65 years, ASA I–II, BMI 18–30) limits generalizability to higher-risk endoscopy patients.

**********

7. PLOS authors have the option to publish the peer review history of their article (what does this mean?). If published, this will include your full peer review and any attached files.

If you choose “no”, your identity will remain anonymous but your review may still be made public.

**Do you want your identity to be public for this peer review?** For information about this choice, including consent withdrawal, please see our Privacy Policy.

Reviewer #1: No

Reviewer #2: No

**********

[NOTE: If reviewer comments were submitted as an attachment file, they will be attached to this email and accessible via the submission site. Please log into your account, locate the manuscript record, and check for the action link "View Attachments". If this link does not appear, there are no attachment files.]

To ensure your figures meet our technical requirements, please review our figure guidelines: https://journals.plos.org/plosone/s/figures

You may also use PLOS’s free figure tool, NAAS, to help you prepare publication quality figures: https://journals.plos.org/plosone/s/figures#loc-tools-for-figure-preparation.

NAAS will assess whether your figures meet our technical requirements by comparing each figure against our figure specifications.

# Author comment: Author response to Decision Letter 1

**DOI:** 10.1371/journal.pone.0350274.r002

21 Feb 2026

Dear Editor,

We would like to thank you and the reviewers for the thorough and constructive feedback on our manuscript entitled " Effect of ciprofol–etomidate mixtures for deep sedation during gastrointestinal endoscopy: Protocol for a three-arm, double-blind randomized controlled trial " (Manuscript ID: PONE-D-25-50283). The comments have been very helpful in improving the clarity and rigor of our study protocol. We have carefully addressed each point raised and have revised the manuscript accordingly. Below is our point-by-point response to the reviewers' comments.

Response to Reviewer 1

Comment 1: In this study protocol authors hypothesized that Gusu Constraint Standing Training (GCST) optimizes gait outcome in hemiparetic stroke patients by enhancing their postural adaptation criticality and improving gait control through the common core pathways and nodes shared by posture and gait. But they didn't specify the hypothesized effect sizes. It is thus not clear to me how they managed to calculate the desired sample sizes in this study. Will lost to follow-up be taken into consideration? Currently you designed 7 times points and some initially included patients may not come back so many times. Would the recruitment of 60 hemiparetic stroke patients be sufficient to achieve a meaningful discovery?

Response: We sincerely thank the reviewer for their time and thoughtful evaluation of our work. After careful review, we suspect there may have been a minor misunderstanding—possibly the comment pertains to another manuscript—as the content regarding GCST and gait outcomes does not align with our current study, which focuses on sedation regimens for gastrointestinal endoscopy. We are encouraged by the positive remarks in the ‘Responses to Questions’ section and have addressed the reviewer’s points to enhance the quality of our manuscript.

Response to Reviewer 2

Comment 1: The primary endpoint combines events with very different clinical importance (e.g., injection pain, myoclonus, airway maneuvers, hypoxemia, hemodynamic changes). This can make the study results hard to interpret clinically. Please explain why a composite is appropriate and provide a clear rationale for including each component. Consider focusing on more clinically meaningful safety outcomes (e.g., hypoxemia requiring interventions, hypotension requiring vasopressors).

Response: We agree with the reviewer's insightful comment. We have gratefully accepted the reviewer's suggestion to refine our primary endpoint by focusing on more clinically meaningful safety outcomes. Accordingly, injection pain, myoclonus, and postoperative nausea and vomiting have now been reclassified as secondary outcomes. This revision enhances the clinical interpretability of our study findings. This revision has been incorporated into the Outcomes section (Page 8–9, Lines 157–182).

Comment 2: The sample size calculation is based on a very small pilot (n=10 per group), which is unstable and may overestimate the effect. I suggest including more patients in the pilot observation or citing published studies. Also, please specify how type I error will be controlled across multiple pairwise comparisons.

Response: We thank the reviewer for this valuable comment. Given the absence of comparable prior studies, we extended the pilot phase over the past month and increased the sample size per group to 30 (including the initial 10 per group). This expanded pilot provides more robust and convincing data. Based on the updated pilot results and the newly defined primary outcome, we recalculated the required sample size.

In the pilot, the composite incidence of adverse events in Groups C, M1, and M2 was 70%, 10%, and 37%, respectively. Using multiple tests for two proportions (rather than the Chi-square test initially considered), which helps control the Type I error, the maximum required sample size per group was calculated to be 38. Accounting for a 20% dropout rate, the total sample size required is 138 participants—only one more per group than our original estimate. We plan to retain the target sample size of 135. After the last patient completes the trial, we will calculate the actual dropout rate. If the number of participants eligible for final analysis exceeds 114, we will not re-randomize or adjust the grouping. This information has been added to the Sample size estimation section (Page 9, Lines 188–191).

Comment 3: Etomidate-associated myoclonus can lead to unblinding, potentially influencing dosing decisions and airway/hemodynamic interventions. The protocol indicates propofol rescue if myoclonus affects the procedure. However, this altered exposure may directly influence outcomes. Thus, blinding may be compromised and rescue-medication rules may bias outcomes.

Response: The reviewer raises a valid methodological concern. The use of etomidate alone for anesthesia induction is associated with a high incidence of myoclonus, whereas combining it with propofol can reduce this adverse effect—this was the rationale behind our design of using a ciprofol–etomidate mixture for total intravenous anesthesia. Although our protocol specified that propofol would be administered as rescue if myoclonus occurred, in our pilot study, no case of severe myoclonus interfered with the completion of gastrointestinal endoscopy.

However, should such a situation arise in the main trial, we plan to include only data collected prior to the rescue intervention (e.g., hypotension, hypoxemia, jaw thrust) in the primary analysis. Data obtained after rescue will be excluded from the primary outcome assessment, as we agree with the reviewer that altered drug exposure may directly influence outcomes. Nonetheless, the incidence of rescue therapy will be recorded and analyzed separately in the final manuscript.

Comment 4: The intervention is defined by volume ratios (1:1 and 2:1) without sufficient details. Please provide a detailed preparation procedure (who prepares, aseptic technique, mixing method, labeling, time limits, temperature/light conditions, disposal). Also, report the final concentrations/doses delivered per mL.

Response: We thank the reviewer for this valuable suggestion. We acknowledge that this aspect was not clearly described in the original manuscript. In response, we have now added a detailed preparation procedure to the Methods section (Page 6, Lines 126–130). The final concentrations and doses delivered per mL was described in Page 8, Lines 123–126.

Comment 5: The restrictive population (age 18–65 years, ASA I–II, BMI 18–30) limits generalizability to higher-risk endoscopy patients.

Response: Thank you very much for this valuable suggestion. Focusing on higher-risk endoscopy patients is indeed a clinically important issue that warrants further investigation. Our current study focuses on the effects of a ciprofol–etomidate mixture on respiratory and circulatory depression, as well as adverse events such as injection pain and myoclonus, in a general population aged 18–65 years, with ASA physical status I–II and BMI 18–30. In future research, we plan to explore the impact of this drug combination in patients at high risk of respiratory complications or in older populations. We have added a statement to this effect in the Limitations of the study design section (Page 14, Lines 305–308).

________________________________________

We hope that our revisions and responses adequately address the reviewers' concerns. We believe the manuscript has been significantly improved as a result, and we are grateful for the opportunity to revise our work. We look forward to your further consideration.

Thank you for your time and guidance.

Yours sincerely,

Jiguo Si

sima225@qq.com

Attachment Submitted filename: Response to Reviewers.docx

# Peer review: Decision Letter 1

**DOI:** 10.1371/journal.pone.0350274.r003

**Authors:** Siraj Ahmed Ali

14 Apr 2026

PONE-D-25-50283R1

Effect of ciprofol–etomidate mixtures for deep sedation during gastrointestinal endoscopy: Protocol for a three-arm, double-blind randomized controlled trial

PLOS One

Dear Dr. Si,

Thank you for submitting your manuscript to PLOS ONE. After careful consideration, we feel that it has merit but does not fully meet PLOS ONE’s publication criteria as it currently stands. Therefore, we invite you to submit a revised version of the manuscript that addresses the points raised during the review process.

Please submit your revised manuscript by May 29 2026 11:59PM. If you will need more time than this to complete your revisions, please reply to this message or contact the journal office at plosone@plos.org. When you're ready to submit your revision, log on to https://www.editorialmanager.com/pone/ and select the 'Submissions Needing Revision' folder to locate your manuscript file.

Please include the following items when submitting your revised manuscript:

- A letter that responds to each point raised by the academic editor and reviewer(s). You should upload this letter as a separate file labeled 'Response to Reviewers'.
- A marked-up copy of your manuscript that highlights changes made to the original version. You should upload this as a separate file labeled 'Revised Manuscript with Track Changes'.
- An unmarked version of your revised paper without tracked changes. You should upload this as a separate file labeled 'Manuscript'.

If you would like to make changes to your financial disclosure, please include your updated statement in your cover letter. Guidelines for resubmitting your figure files are available below the reviewer comments at the end of this letter.

If applicable, we recommend that you deposit your laboratory protocols in protocols.io to enhance the reproducibility of your results. Protocols.io assigns your protocol its own identifier (DOI) so that it can be cited independently in the future. For instructions see: https://journals.plos.org/plosone/s/submission-guidelines#loc-laboratory-protocols. Additionally, PLOS ONE offers an option for publishing peer-reviewed Lab Protocol articles, which describe protocols hosted on protocols.io. Read more information on sharing protocols at https://plos.org/protocols?utm_medium=editorial-email&utm_source=authorletters&utm_campaign=protocols.

As the corresponding author, your ORCID iD is verified in the submission system and will appear in the published article. PLOS supports the use of ORCID, and we encourage all coauthors to register for an ORCID iD and use it as well. Please encourage your coauthors to verify their ORCID iD within the submission system before final acceptance, as unverified ORCID iDs will not appear in the published article. Only the individual author can complete the verification step; PLOS staff cannot verify ORCID iDs on behalf of authors.

We look forward to receiving your revised manuscript.

Kind regards,

Johanna Pruller

Senior Editor

PLOS One

on behalf of

Siraj Ahmed Ali

Academic Editor

PLOS One

Journal Requirements:

If the reviewer comments include a recommendation to cite specific previously published works, please review and evaluate these publications to determine whether they are relevant and should be cited. There is no requirement to cite these works unless the editor has indicated otherwise.

[Note: HTML markup is below. Please do not edit.]

Reviewers' comments:

Reviewer's Responses to Questions

**Comments to the Author**

1. Does the manuscript provide a valid rationale for the proposed study, with clearly identified and justified research questions?

The research question outlined is expected to address a valid academic problem or topic and contribute to the base of knowledge in the field.

Reviewer #1: Yes

**********

2. Is the protocol technically sound and planned in a manner that will lead to a meaningful outcome and allow testing the stated hypotheses?

The manuscript should describe the methods in sufficient detail to prevent undisclosed flexibility in the experimental procedure or analysis pipeline, including sufficient outcome-neutral conditions (e.g. necessary controls, absence of floor or ceiling effects) to test the proposed hypotheses and a statistical power analysis where applicable. As there may be aspects of the methodology and analysis which can only be refined once the work is undertaken, authors should outline potential assumptions and explicitly describe what aspects of the proposed analyses, if any, are exploratory.

Reviewer #1: Yes

**********

3. Is the methodology feasible and described in sufficient detail to allow the work to be replicable?

Descriptions of methods and materials in the protocol should be reported in sufficient detail for another researcher to reproduce all experiments and analyses. The protocol should describe the appropriate controls, sample size calculations, and replication needed to ensure that the data are robust and reproducible.

Reviewer #1: Yes

**********

4. Have the authors described where all data underlying the findings will be made available when the study is complete?

The PLOS Data policy requires authors to make all data underlying the findings described in their manuscript fully available without restriction, with rare exception, at the time of publication. The data should be provided as part of the manuscript or its supporting information, or deposited to a public repository. For example, in addition to summary statistics, the data points behind means, medians and variance measures should be available. If there are restrictions on publicly sharing data—e.g. participant privacy or use of data from a third party—those must be specified.

Reviewer #1: Yes

**********

5. Is the manuscript presented in an intelligible fashion and written in standard English?

PLOS ONE does not copyedit accepted manuscripts, so the language in submitted articles must be clear, correct, and unambiguous. Any typographical or grammatical errors should be corrected at revision, so please note any specific errors here.

Reviewer #1: Yes

**********

6. Review Comments to the Author

Please use the space provided to explain your answers to the questions above and, if applicable, provide comments about issues authors must address before this protocol can be accepted for publication. You may also include additional comments for the author, including concerns about research or publication ethics.

You may also provide optional suggestions and comments to authors that they might find helpful in planning their study.

(Please upload your review as an attachment if it exceeds 20,000 characters)

Reviewer #1: This protocol intended to study the safety and efficacy of two ciprofol-etomidate mixtures at different volume ratios compared with ciprofol alone in patients with undergoing gastrointestinal endoscopy.

A three arm follow-up study will be conducted with 135 patients. The sample size calculation is based on an initial pilot data set obtained from the similar population. However, it seems you didn’t adjust for loss to follow up.

How the ratio of 1:1 and 2:1 is determined in this study?

The discussion on safety management is limited. How do you find a trade-off between toxicity and efficacy?

The main outcome is the AE incidence. However, in your statistical analysis section you didn’t discuss how to model the treatment effects on this clinical outcome. Are you going to use logistic regression and report odds ratio? Or do you need to use some longitudinal data analysis approaches since you have repeated measures? You mentioned repeated measures ANOVA in this section but this approach may not be suitable for AE incidence.

Page 3, there is a typesetting error at line 58.

**********

7. PLOS authors have the option to publish the peer review history of their article (what does this mean?). If published, this will include your full peer review and any attached files.

If you choose “no”, your identity will remain anonymous but your review may still be made public.

**Do you want your identity to be public for this peer review?** For information about this choice, including consent withdrawal, please see our Privacy Policy.

Reviewer #1: No

**********

[NOTE: If reviewer comments were submitted as an attachment file, they will be attached to this email and accessible via the submission site. Please log into your account, locate the manuscript record, and check for the action link "View Attachments". If this link does not appear, there are no attachment files.]

To ensure your figures meet our technical requirements, please review our figure guidelines: https://journals.plos.org/plosone/s/figures

You may also use PLOS’s free figure tool, NAAS, to help you prepare publication quality figures: https://journals.plos.org/plosone/s/figures#loc-tools-for-figure-preparation.

NAAS will assess whether your figures meet our technical requirements by comparing each figure against our figure specifications.

# Author comment: Author response to Decision Letter 2

**DOI:** 10.1371/journal.pone.0350274.r004

27 Apr 2026

Dear Editor,

We would like to thank you and the reviewers for the thorough and constructive feedback on our manuscript entitled " Effect of ciprofol–etomidate mixtures for deep sedation during gastrointestinal endoscopy: Protocol for a three-arm, double-blind randomized controlled trial " (Manuscript ID: PONE-D-25-50283). The comments have been very helpful in improving the clarity and rigor of our study protocol. We have carefully addressed each point raised and have revised the manuscript accordingly. Below is our point-by-point response to the reviewer's comments.

Response to Reviewer 1

Comment 1: A three-arm follow-up study will be conducted with 135 patients. The sample size calculation is based on an initial pilot data set obtained from the similar population. However, it seems you didn’t adjust for loss to follow up.

Response: Thank you for your question. Due to the lack of prior data for reference, we first conducted a pilot study with a small sample size (10 patients per group, 30 in total). A previous reviewer expressed concern about the limited sample size. We have taken this feedback seriously and decided to expand the sample size to 30 patients per group (including the initial 10 per group). Finally, the composite incidence of adverse events in Groups C, M1, and M2 was 70%, 10%, and 37%, respectively. Using multiple tests for two proportions (rather than the Chi-square test initially considered), the maximum required sample size per group was calculated to be 38. Accounting for a 20% dropout rate, this yields 46 patients per group—only one more than our original calculation. Since the study is currently ongoing and randomization has already been performed, we determined not to restart the study. Instead, we will continue enrollment and ensure that at least 38 evaluable patients are obtained per group by the end of the study. The final analysis and report will then be conducted based on these complete data.

Comment 2: How the ratio of 1:1 and 2:1 is determined in this study?

Response: We thank the reviewer for this valuable comment. Both ciprofol and etomidate act on GABA-A receptors but differ in their potency, pharmacokinetic profiles, and side effects (e.g., injection pain, myoclonus, respiratory depression). Ratios of 1:1 and 2:1 ratio were empirically selected for this preliminary study to compare two fixed-volume mixtures. As this is an initial exploratory protocol, we did not aim to determine the optimal ratio. We have now added a statement in the Discussion acknowledging that further dose-finding studies are needed to establish the best ratio.

Comment 3: The discussion on safety management is limited. How do you find a trade-off between toxicity and efficacy?

Response: We thank the reviewer for this insightful and important comment. We agree that the original manuscript did not sufficiently discuss how we balance efficacy and toxicity in the study design. In response, we have added a new paragraph in the Discussion section (page 14, line 299-300 and line 303-312) to explicitly address this trade-off. In this newly added part, we clarify as follow.

1. Complementary pharmacology: Ciprofol provides deep sedation with low injection pain but causes hypotension, whereas etomidate offers hemodynamic stability but induces myoclonus and nausea. By combining them in two volume‑to‑volume ratios (1:1 and 2:1), we aim to retain the advantages of each while minimizing their respective toxicities.

2. Composite primary outcome: The use of a composite endpoint (including hypotension, hypoxemia, myoclonus, injection pain, nausea/vomiting, etc.) reflects overall safety rather than a single toxicity, allowing a balanced assessment of net clinical benefit.

3. Rescue and discontinuation rules: Predefined criteria for top‑up doses and alternative sedation (propofol) prevent prolonged exposure to an ineffective or unsafe regimen. Explicit protocols for managing hypotension, bradycardia, hypoxemia, myoclonus and nausea ensure patient safety without compromising the evaluation of the study drugs.

Comment 4: The main outcome is the AE incidence. However, in your statistical analysis section you didn’t discuss how to model the treatment effects on this clinical outcome. Are you going to use logistic regression and report odds ratio? Or do you need to use some longitudinal data analysis approaches since you have repeated measures? You mentioned repeated measures ANOVA in this section but this approach may not be suitable for AE incidence.

Response: We thank the reviewer for this rigorous and valuable comment. We have revised the Statistical analysis section in the manuscript (page 12, line 251-253) as follows: For the primary outcome—the composite incidence of adverse events—a binary logistic regression model will be used to compare the three groups. Odds ratios with 95% confidence intervals will be reported.

In our original Statistical methods section, we stated that repeated‑measures ANOVA would be employed “for vital signs” rather than primary outcome, there appears to be a misunderstanding regarding the intended use of repeated‑measures ANOVA in our manuscript.

Comment 5: Page 3, there is a typesetting error at line 58.

Response: We thank the reviewer for pointing out the typesetting error. We have now changed British spelling to American English accordingly. We greatly appreciate the reviewer's careful reading and helpful suggestion.

________________________________________

We hope that our revisions and responses adequately address the reviewer’s concerns. We believe the manuscript has been significantly improved as a result, and we are grateful for the opportunity to revise our work. We look forward to your further consideration.

Thank you for your time and guidance.

Yours sincerely,

Jiguo Si

sima225@qq.com

Attachment Submitted filename: Response_to_Reviewers_auresp_2.docx

# Editor report: Decision Letter 2

**DOI:** 10.1371/journal.pone.0350274.r005

**Authors:** Siraj Ahmed Ali

13 May 2026

Effect of ciprofol–etomidate mixtures for deep sedation during gastrointestinal endoscopy: Protocol for a three-arm, double-blind randomized controlled trial

PONE-D-25-50283R2

Dear Dr. Jiguo Si,

We’re pleased to inform you that your manuscript has been judged scientifically suitable for publication and will be formally accepted for publication once it meets all outstanding technical requirements.

Within one week, you’ll receive an e-mail detailing the required amendments. When these have been addressed, you’ll receive a formal acceptance letter and your manuscript will be scheduled for publication.

An invoice will be generated when your article is formally accepted. Please note, if your institution has a publishing partnership with PLOS and your article meets the relevant criteria, all or part of your publication costs will be covered. Please make sure your user information is up-to-date by logging into Editorial Manager at Editorial Manager® and clicking the ‘Update My Information' link at the top of the page. For questions related to billing, please contact billing support.

If your institution or institutions have a press office, please notify them about your upcoming paper to help maximize its impact. If they’ll be preparing press materials, please inform our press team as soon as possible -- no later than 48 hours after receiving the formal acceptance. Your manuscript will remain under strict press embargo until 2 pm Eastern Time on the date of publication. For more information, please contact onepress@plos.org.

Kind regards,

Siraj Ahmed Ali

Academic Editor

PLOS One

Additional Editor Comments (optional):

Reviewers' comments:

# Editor report: Acceptance letter

**DOI:** 10.1371/journal.pone.0350274.r006

**Authors:** Siraj Ahmed Ali

PONE-D-25-50283R2

PLOS One

Dear Dr. Si,

I'm pleased to inform you that your manuscript has been deemed suitable for publication in PLOS One. Congratulations! Your manuscript is now being handed over to our production team.

At this stage, our production department will prepare your paper for publication. This includes ensuring the following:

* All references, tables, and figures are properly cited

* All relevant supporting information is included in the manuscript submission,

* There are no issues that prevent the paper from being properly typeset

You will receive further instructions from the production team, including instructions on how to review your proof when it is ready. Please keep in mind that we are working through a large volume of accepted articles, so please give us a few days to review your paper and let you know the next and final steps.

Lastly, if your institution or institutions have a press office, please let them know about your upcoming paper now to help maximize its impact. If they'll be preparing press materials, please inform our press team within the next 48 hours. Your manuscript will remain under strict press embargo until 2 pm Eastern Time on the date of publication. For more information, please contact onepress@plos.org.

You will receive an invoice from PLOS for your publication fee after your manuscript has reached the completed accept phase. If you receive an email requesting payment before acceptance or for any other service, this may be a phishing scheme. Learn how to identify phishing emails and protect your accounts at https://explore.plos.org/phishing.

If we can help with anything else, please email us at customercare@plos.org.

Thank you for submitting your work to PLOS ONE and supporting open access.

Kind regards,

PLOS ONE Editorial Office Staff

on behalf of

Dr. Siraj Ahmed Ali

Academic Editor

PLOS One
